# Supplementary material for: Restoration of visual function in advanced disease after transplantation of purified human pluripotent stem cell-derived cone photoreceptors
Source: Cell Rep. 2021 Apr 20;35(3):109022. doi: 10.1016/j.celrep.2021.109022 (PMC8065177; doi:10.1016/j.celrep.2021.109022)
Supplement: Document S1. Figures S1–S7 and Tables S1 and S2 [file mmc1.pdf]

Cell Reports, Volume *35*

Supplemental information

Restoration of visual function in advanced disease after transplantation of purified human pluripotent stem cell-derived cone photoreceptors

Joana Ribeiro, Christopher A. Procyk, Emma L. West, Michelle O'Hara-Wright, Monica F. Martins, Majid Moshtagh Khorasani, Aura Hare, Mark Basche, Milan Fernando, Debbie Goh, Neeraj Jumbo, Matteo Rizzi, Kate Powell, Menahil Tariq, Michel Michaelides, James W.B. Bainbridge, Alexander J. Smith, Rachael A. Pearson, Anai Gonzalez-Cordero, and Robin R. Ali

SUPPLEMENTARY INFORMATION

Supplementary Figures

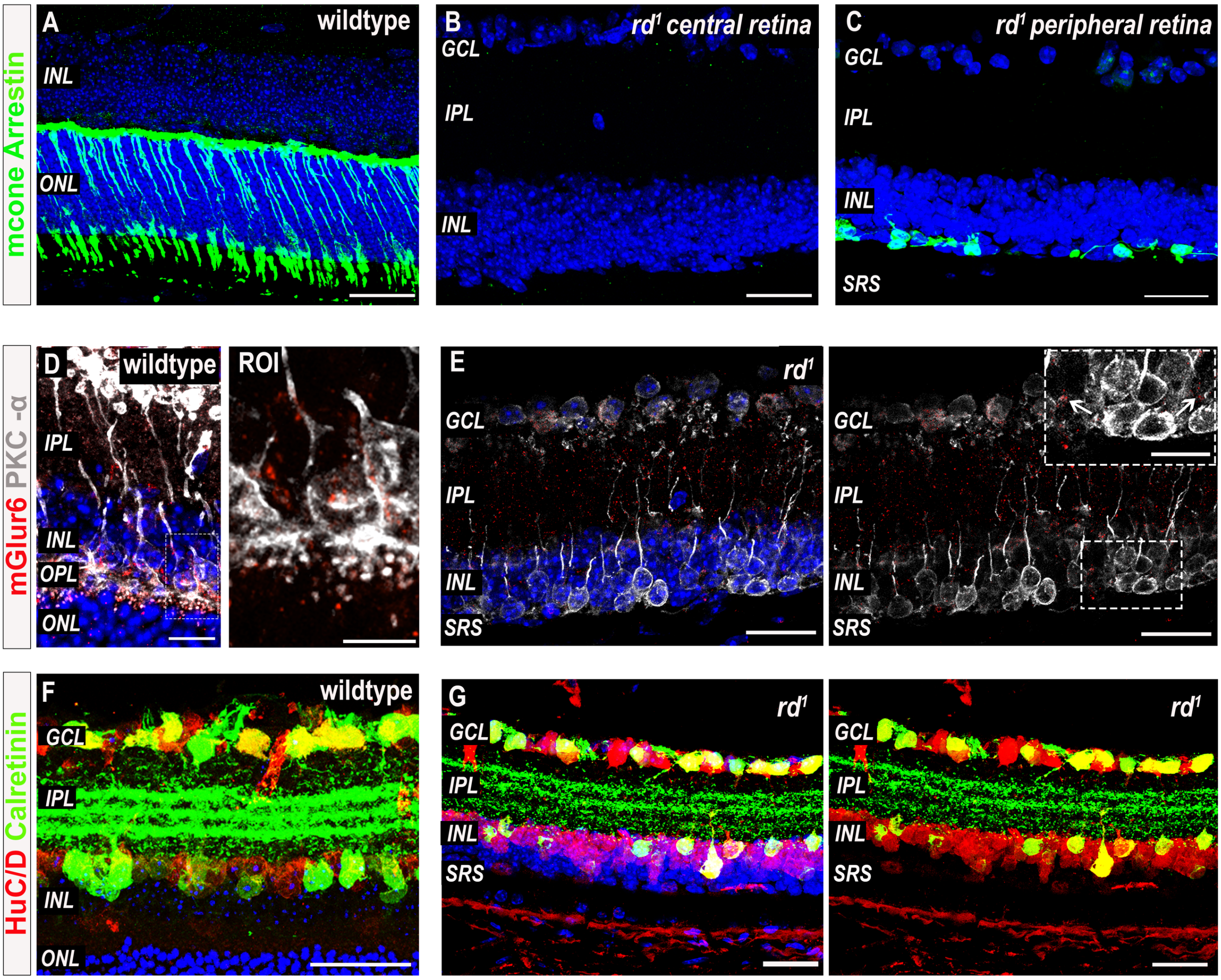

Supplementary Figure S1. Characterization of 3-month old *rd1/Foxn1^{nu}* retina. Related to Figures 1 and 3 Representative confocal images of immunohistochemistry for retinal cell markers (N ≥ 3 animals). **(A)** 3-month old wildtype retina showing typical cone morphology. **(B)** Age-matched *rd^{1}/FoxN1^{nu}* (*rd^{1}*) retina. The majority of the retina did not show presence of any cone photoreceptor cells. Image shows central retina. **(C)** Small, typically peripheral, regions of the *rd^{1}* retina retained a few disorganized Cone Arrestin+ (*green*) cones. Image shows mid-peripheral retina. **(D)** Wildtype retinas display widespread punctate labelling for mGluR6 (*red*) in the OPL. This colocalised with the PKC-a+ (*grey*) post-synaptic processes of rod bipolar cells (ROI, *right*). **(E)** In the *rd^{1}*, bipolar cells displayed a significantly reduced dendritic arborization. While some mis-localized mGluR6 staining was visible in the IPL, little or no mGluR6 positive structures were found between the INL and SRS (insert). **(F)** Wildtype retina showing morphology and distribution of amacrine and ganglion cells in the retina. **(G)** In the *rd^{1}*, amacrine cells had unusual morphology, as they appeared fewer and smaller. Some disorganization and reduction in staining for calretinin positive processes was visible in the IPL. Ganglion cells displayed typical morphology. Scale bar 25µm, except inner panel in E, 12.5 µm). GCL – ganglion cell layer, IPL – inner plexiform layer, INL – inner nuclear layer, OPL – outer plexiform later, ONL – outer nuclear layer, SRS – subretinal space).

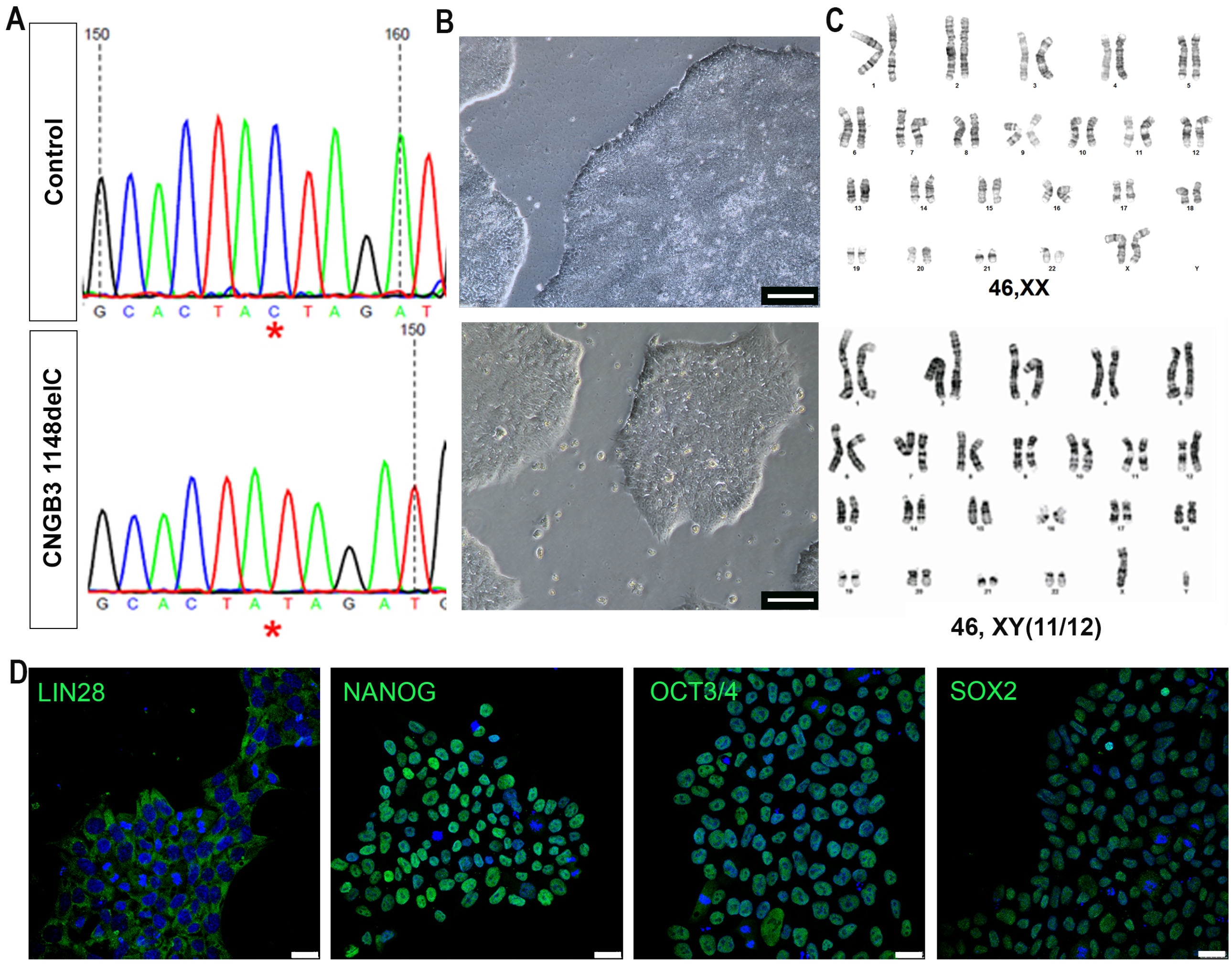

Supplementary Figure S2. Characterisation of CNGB3 hiPSC line. Related to Figure 1. PBMCs from CNGB3 patient was reprogrammed using episomal methods. **(A)** Sequencing data of the WT hESC line and the CNGB3 hiPSC line showing the existence of a single base pair deletion in the CNGB3 hiPSC line (*red asterisk*). **(B)** Representative bright field image of both cell lines showing typical pluripotent cell colonies morphology. **(C)** A normal karyotype was observed in both lines. **(D)** Representative examples of immunostaining showing that the CNGB3 cell line was positive for all pluripotency markers tested. Scale bars 100µm, B; 25µm, D.

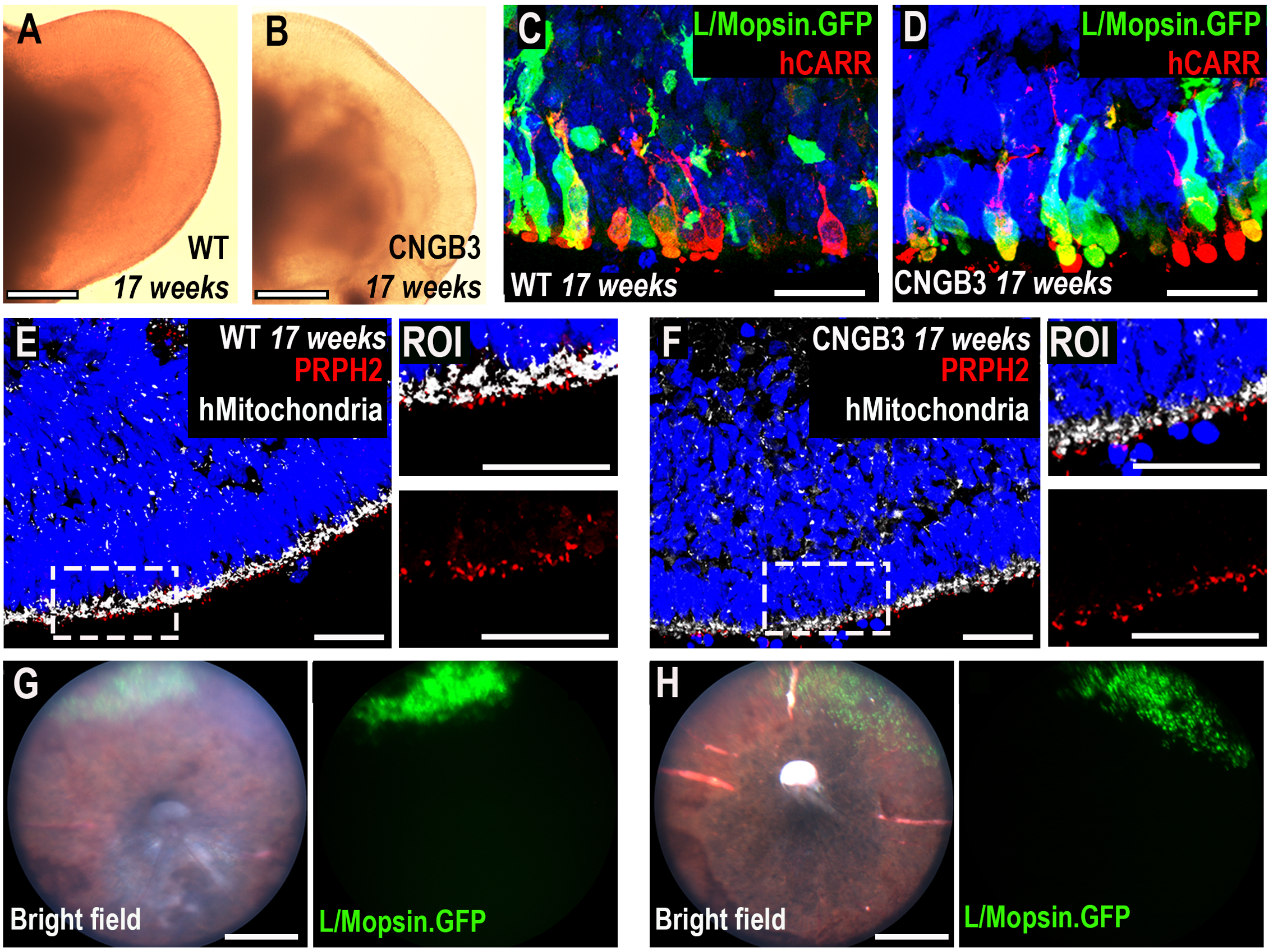

Supplementary Figure S3. Differentiation and transplantation of cone photoreceptors from WT and CNGB3 PSC lines. Related to Figures 1 and 2. **(A)** Representative bright-field image of WT hESC-derived retinal organoid at week 17 of differentiation. **(B)** Representative bright-field image of CNGB3 hiPSC-derived retinal organoid at week 17 of differentiation displaying very similar morphology. **(C, D)** Human CONE ARRESTIN+ (*red*) cells were detected by 17 weeks of differentiation and displayed a similar morphology in WT and CNGB3 derived organoids. **(E, F)** At 17 weeks, organoids derived from both cell lines show presence of a mitochondria-enriched (*grey*) IS-like region. Small PRPH2+ (*red*) buds were present in both WT and CNGB3 derived organoids and these were correctly located distal to mitochondria-rich IS. **(G-H)** Representative examples of L/MOpsin GFP+ cones in both WT **(G)** and CNGB3 **(H)** organoids. **(I)** proportion of total cells that were GFP+ at 17weeks of differentiation in organoids derived from WT and CNGB3 cell lines, as determined by FACS. **(J-K)** Representative fundoscopy images showing that, following transplantation, the GFP+ WT cones **(J)** and CNGB3 cones **(K)** survive similarly in the subretinal space, up to at least 12 weeks post- transplantation. **(L)** Low power montage showing extent of spread of transplanted GFP+ WT cones. Scale bars, 70µm, A-B; 25µm, C-F; 75µm, J-K.

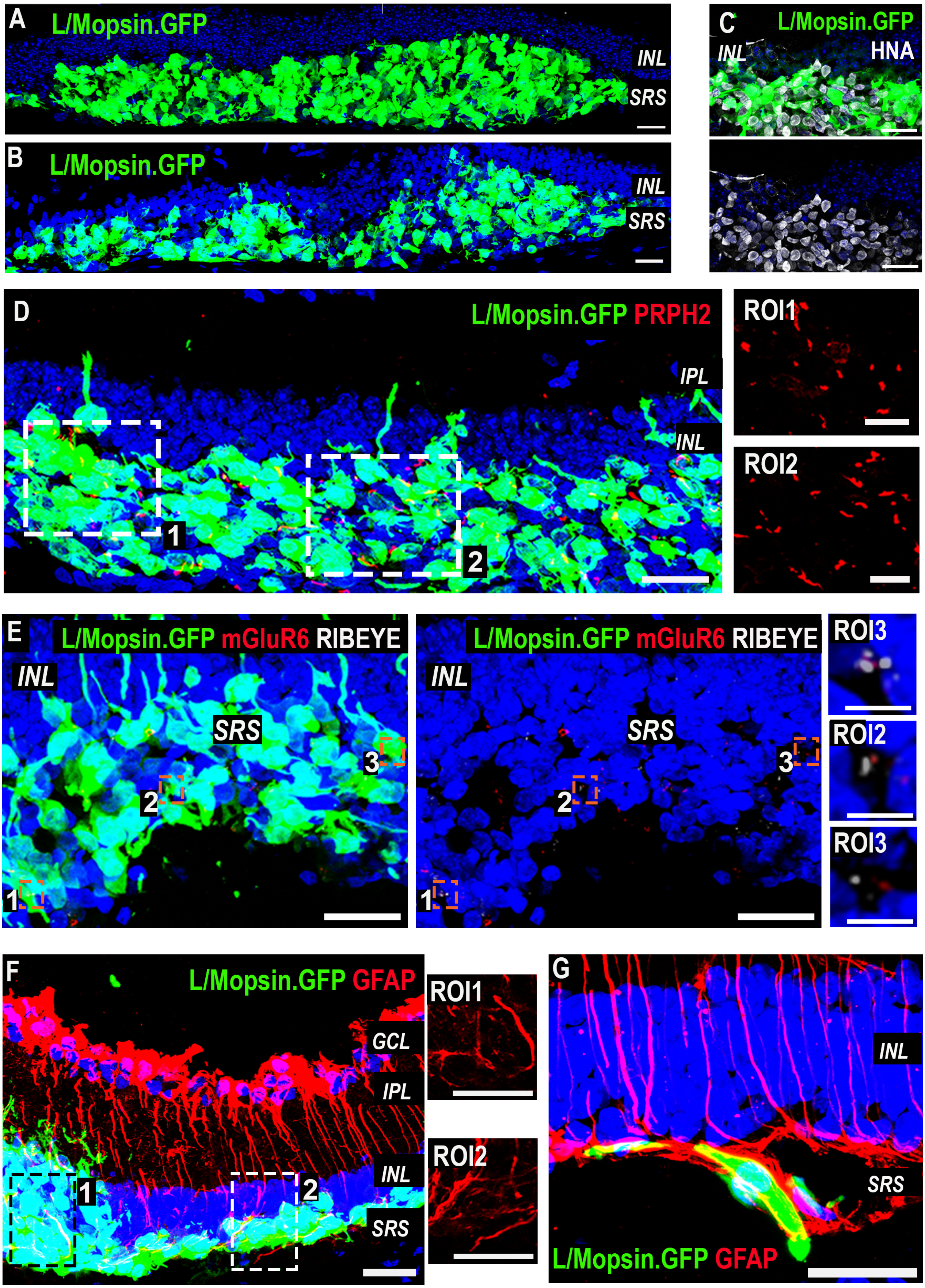
A
L/Mopsin.GFP
INL
SRS
B
L/Mopsin.GFP
INL
SRS
C
L/Mopsin.GFP
HNA
INL
D
L/Mopsin.GFP PRPH2
IPL
INL
1
2
ROI1
ROI2
E
L/Mopsin.GFP mGluR6 RIBEYE
INL
SRS
1
2
3
L/Mopsin.GFP mGluR6 RIBEYE
INL
SRS
1
2
3
ROI3
ROI2
ROI3
F
L/Mopsin.GFP GFAP
GCL
IPL
INL
SRS
1
2
ROI1
ROI2
G
INL
SRS
L/Mopsin.GFP GFAP

Supplementary Figure S4. Transplanted WT and CNGB3 cones mature in the *rd1* retina. Related to Figures 1-3. Representative confocal images of immunohistochemistry for retinal cell markers. Representative confocal images of immunohistochemistry for retinal cell markers. **(A, B)** Representative images of dense GFP+ cell mass following transplantation of **(A)** WT- and **(B)** CNGB3-derived cones. **(C)** CNGB3 cones survived in the subretinal space (SRS) and expressed human nuclear antigen (HNA; *grey*) (bottom). **(D)** CNGB3 cones matured *in vivo*, presenting PRPH2+ segment-like structures (ROI1, ROI2). **(E)** Pre-synaptic RIBEYE (grey) and post-synaptic mGluR6 (red) proteins were found in close proximity to one another in *rd1* mice transplanted with CNGB3 cones (ROI1-3). **(F, G)** GFAP+ (*red*) host Müller glial processes extended into the cell mass. No glial scar was observed (ROI1, ROI2). Scale bar 25µm, except ROIs in E 6.25µm. GCL – ganglion cell layer, IPL – inner plexiform layer, INL – inner nuclear layer, SRS – subretinal space.

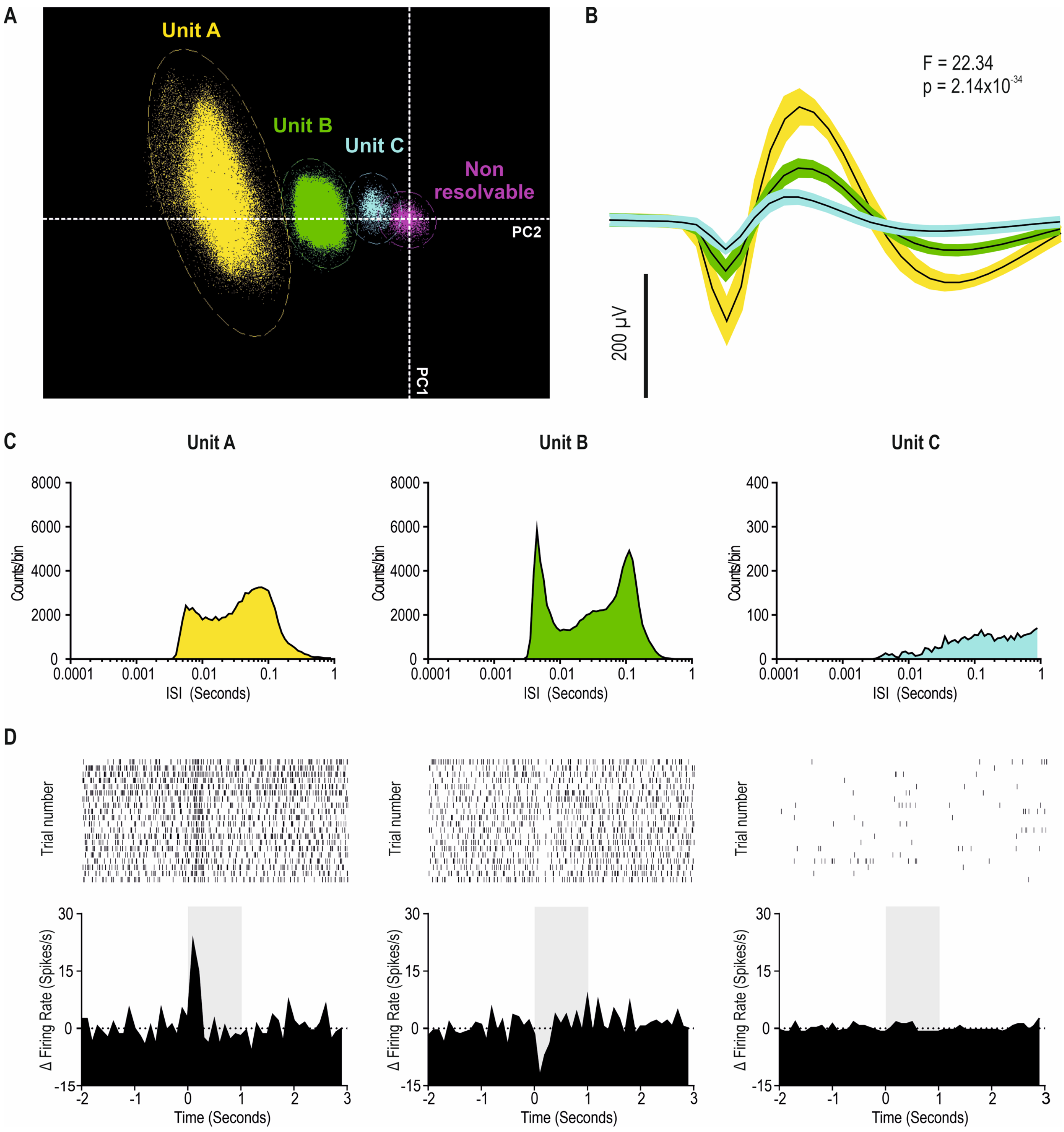

Supplementary Figure S5. Analysis of Multi Electrode Array (MEA) data. Related to Figure 6 **(A)** Spike waveforms in a 2D representation of principal component space from a representative channel from *rd1* retina treated with WT cones (X axis = PC1; Y axis = PC2) shows three identifiable single units (Unit A = Yellow; Unit B = Green; Unit C = Blue; Non-resolvable spikes = Purple). **(B)** Unit A, B and C show differences in spike shape and amplitude (mean ± SD). High Manova F values and low P-values indicate good cluster separation for isolated single units. **(C)** Log Inter-spike Interval histograms of Unit A (yellow), B (green) and C (blue) show peaks at discrete intervals greater than 1ms, indicative of spikes fired in bursts. **(D)** (*top*) Raster plot and *(bottom)* average PSTH show responses of the single units isolated in *(A)*. Unit A =Transient ON, Unit B = Suppressed ON and Unit C = non-light responsive when presented with 20 repeats of a 1s light pulse from darkness and a 10s ISI (grey bar indicates light pulse; irradiance = 4.21×10^{15} photons$\cdot$cm$^{-2}\cdot$s^{-1}).

A

Response Categorisation	Response Profile	*Gnat1*$^{-/-}$	*rd*^{1} + *WT cones*
ON	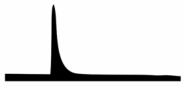	10.3	25.9
On Suppressed by Dark	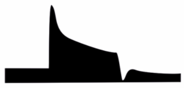	11.3	2.5
ON-OFF	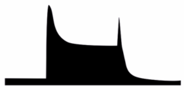	47.8	6.4
Slow Sustained	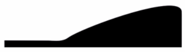	0.0	1.2
OFF	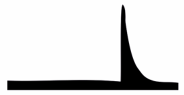	8.7	3.8
OFF Suppressed by Light	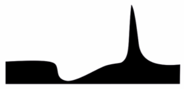	11.9	1.7
Suppressed ON	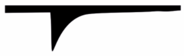	0.9	6.4
Suppressed OFF	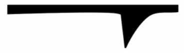	1.4	1.6
Suppressed ON-OFF	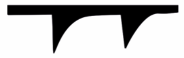	3.8	0.0
Non Light Responsive		3.9	50.5

B

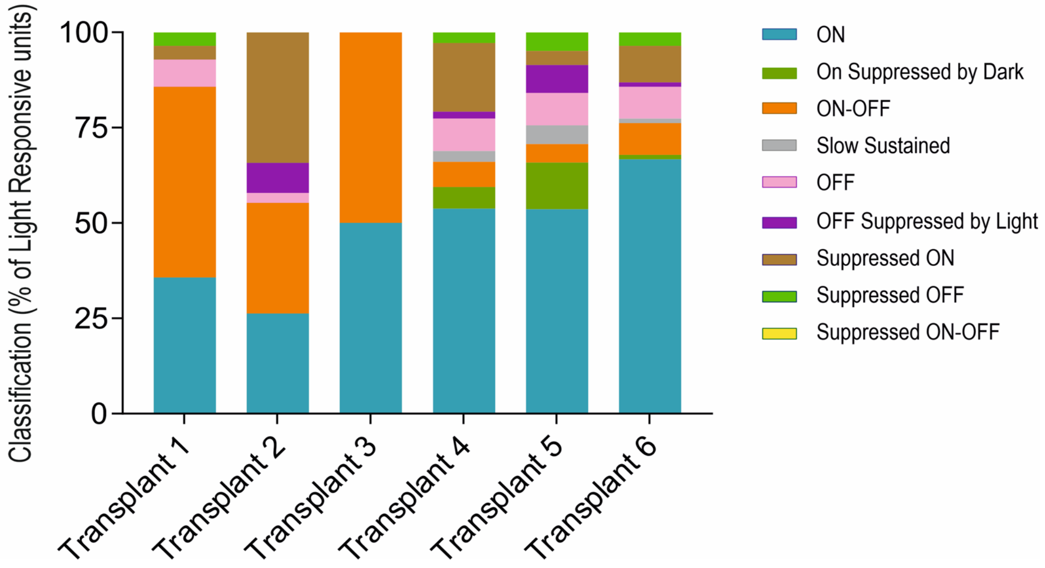

Supplementary Figure S6: Reproducibility of light-responses in *rd1/FoxN1^{nu}* treated with WT cones. Related to Figure 6. **(A)** % (mean) response type of single units for *rd1/FoxN1^{nu} (rd^{1})* retinas transplanted with WT cones (N = 6 retinas; n = 687 single units) and *Gnat1$^{-/-}$* (N = 5; n = 1031) mice. **(B)** A similar variety of light response types were seen in each of the six *rd^{1}* retinas transplanted with WT cones (Total light-responsive units = 340; N = 6).

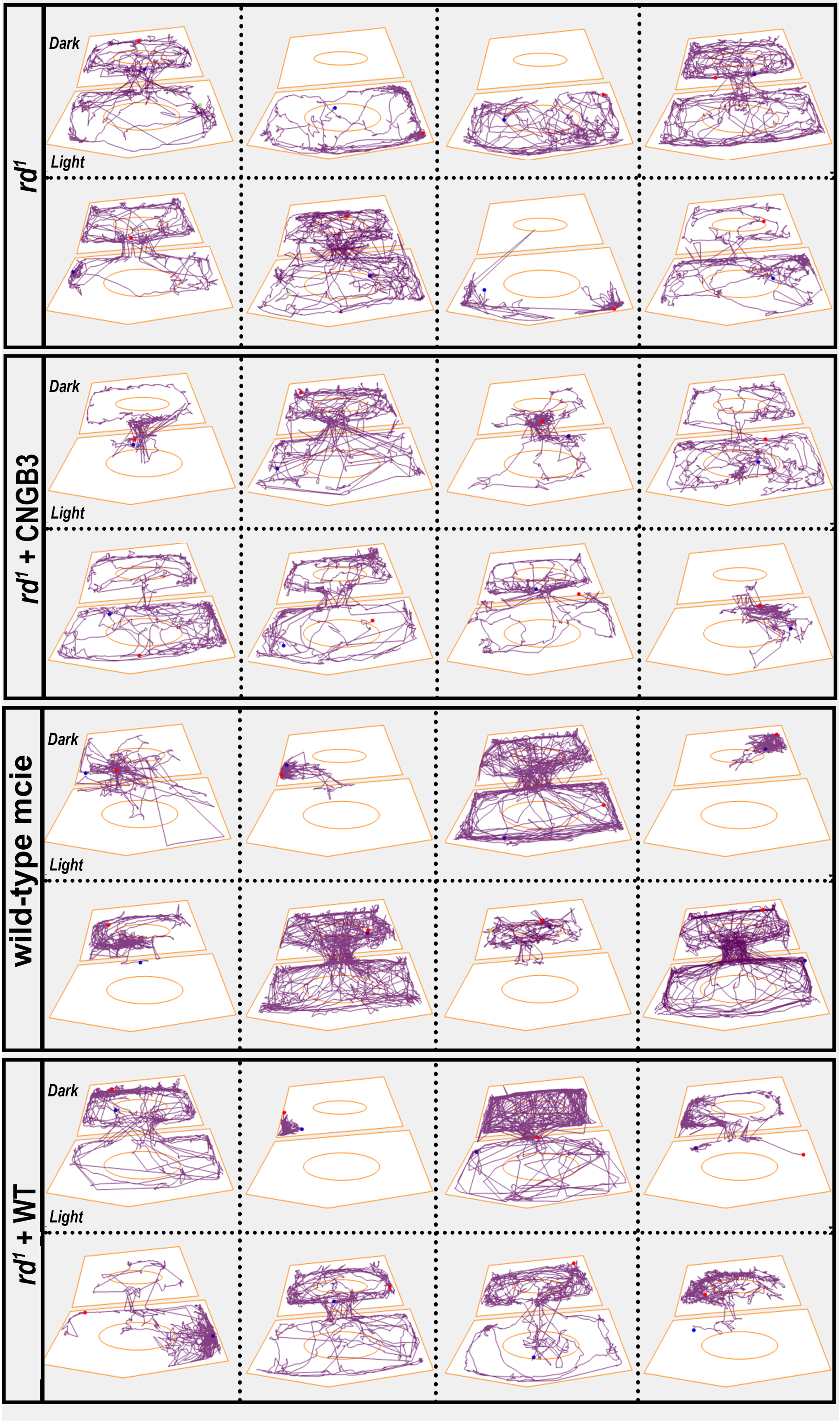
rd1
Dark
Light
rd1 + CNGB3
Dark
Light
wild-type mcie
Dark
Light
rd1 + WT
Dark
Light

Supplementary Figure S7. Transplantation of WT cones restores light avoidance behaviour in *rd*^{1} mice. Related to Figure 7. Further representative examples, chosen randomly, of tracking plots from different animals in each experimental group. Blue dot represents each animal's position at the start of the recording and red dot the position at the end of the recording. Ellipsoids (I-L) define the central area of each compartment. Untreated *rd*^{1} animals and those receiving non-functional CNGB3 cones displayed a similar pattern of movement, with most mice exploring both light and dark compartments equally. In contrast, *rd*^{1} animals transplanted with WT cones showed a clear preference for the dark compartment of the arena. Most *rd*^{1} animals transplanted with WT cones showed similar exploration pattern to normal wildtype animals.

Supplementary Tables

Animal	*Gnat1* $^{-/-}$	*rd*^{1} + WT cones	*rd*^{1} + CNGB3 cones	*rd*^{1}
**Number of recorded retinas**	5	6	5	5
**Transplanted cell line**	N/A	hESC H9	hiPSC CNGB3	N/A
**Number of transplanted cones per retina**	N/A	450-500k	450-500k	N/A
**Mouse age at transplantation**	N/A	3 months	3 months	N/A
**Mouse age at recording**	6 months	6 months	6 months	6 months
**Number of recorded Electrodes**	600	540	480	600
**mERG**	560	150	0	0
**Single Units**	1031	687	945	641
**Light Responsive**	991	340	8	13
**Non Light Responsive**	40	347	937	628

Supplemental Table S1. Summary of MEA recordings in all experimental groups. Related to Figures 4, 5 & 6. Table summarises the key information from Multi Electrode Array recordings in each experimental group: the number of retinas recorded, the age at transplantation (if applicable), the cell line transplanted (if applicable), the number of cells transplanted (if applicable), age of retina at time of recording, number of electrodes recorded, number of electrodes with positive mERG, number of single units resolved after spike sorting, and the number of those units which were light-responsive or non-light responsive following classification.

Response Categorisation	ON	OFF	ON Suppressed	OFF Suppressed	Sustained
**Type 1**<br>ON	1	0	0	0	0
**Type 2**<br>On Suppressed by Dark	1	0	0	1	0
**Type 3**<br>ON-OFF	1	1	0	0	0
**Type 4**<br>Slow Sustained	1	1	0	0	1
**Type 5**<br>OFF	0	1	0	0	0
**Type 6**<br>OFF Suppressed by light	0	1	1	0	0
**Type 7**<br>Suppressed ON	0	0	1	0	0
**Type 8**<br>Suppressed OFF	0	0	0	1	0
**Type 9**<br>Suppressed ON-OFF	0	0	1	1	0
**Type 10**<br>Non light responsive	0	0	0	0	0

Supplementary Table S2. Summary of rules used to classify neural responses into one of 10 categories. Related to STAR Methods, section *Identification of Light Responses*. Single units were defined as light responsive when their firing rate either increased (+3SD) or decreased (-1SD) when compared to the pre-stimulus baseline. The time at which neuronal activity crossed the threshold in response to the 1s light step was defined in three timeframes: 1) Duration of the light stimulus (1s; ON period), 2) Duration of time directly following the light stimulus and equal to it (1s; OFF period), 3) Duration of time beyond the OFF period (Sustained period) which was defined as 6 times the ON period. When single unit responses cross the threshold within the specified frame, the expression value is 1 (True) or 0 (False), and generated a look up table which classified the ten response categories. When the response crosses the threshold in the specified time span, the expression value becomes 1 (True) or 0 (False).
