# Supplementary material for: Restoration of visual function in advanced disease after transplantation of purified human pluripotent stem cell-derived cone photoreceptors
Source: Cell Rep. 2021 Apr 20;35(3):109022. doi: 10.1016/j.celrep.2021.109022 (PMC8065177; doi:10.1016/j.celrep.2021.109022)
Supplement: Document S2. Article plus supplemental information [file mmc2.pdf]

Cell Reports

Article

Restoration of visual function in advanced disease after transplantation of purified human pluripotent stem cell-derived cone photoreceptors

Graphical abstract

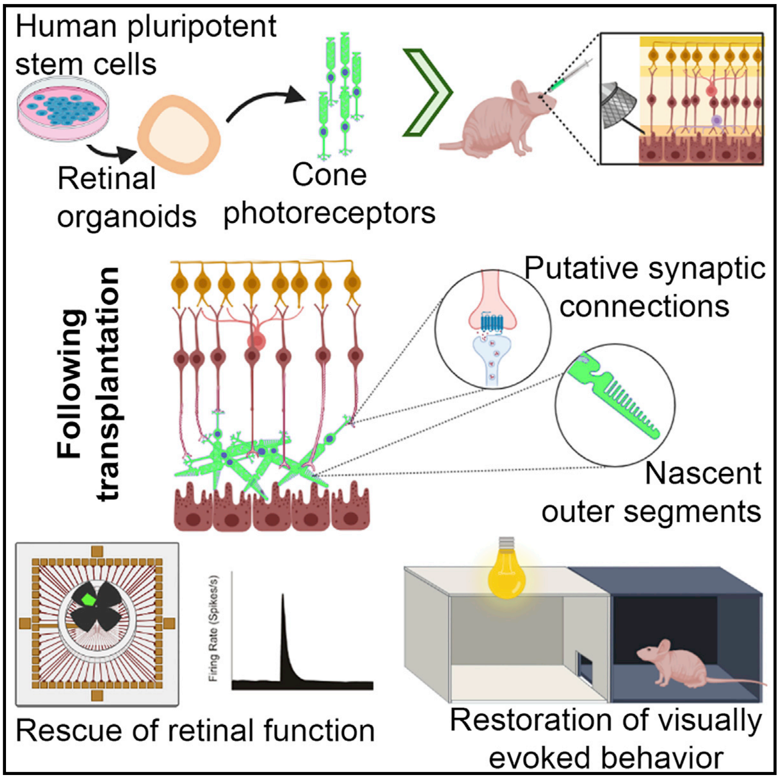

Highlights

- Rescue of cone-mediated function by transplantation of purified human cones
- Restoration of complex retinal responses and behavior in advanced degeneration
- Formation of human-murine putative synaptic connections
- Relevant controls exclude material transfer and trophic support

Authors

Joana Ribeiro, Christopher A. Procyk, Emma L. West, ..., Rachael A. Pearson, Anai Gonzalez-Cordero, Robin R. Ali

Correspondence

robin.ali@kcl.ac.uk

In brief

Cone photoreceptor death and associated central vision loss are common to many retinal dystrophies. Ribeiro et al. show that transplantation of purified human pluripotent stem cell-derived cones into a mouse model of end-stage disease allows the formation of functional connections with the underlying retina and restores cone-mediated visual function.

Ribeiro et al., 2021, Cell Reports *35*, 109022
April 20, 2021 © 2021 The Authors.
https://doi.org/10.1016/j.celrep.2021.109022

CellPress

Cell Reports

CellPress
OPEN ACCESS

Article

Restoration of visual function in advanced disease after transplantation of purified human pluripotent stem cell-derived cone photoreceptors

Joana Ribeiro,[1,3,6] Christopher A. Procyk,[1,4,6] Emma L. West,[1,4] Michelle O'Hara-Wright,[1] Monica F. Martins,[1] Majid Moshtagh Khorasani,[1,4] Aura Hare,[1,4] Mark Basche,[1,4] Milan Fernando,[1] Debbie Goh,[1] Neeraj Jumbo,[1] Matteo Rizzi,[1,4] Kate Powell,[1,4] Menahil Tariq,[1] Michel Michaelides,[1] James W.B. Bainbridge,[1] Alexander J. Smith,[1,4] Rachael A. Pearson,[1,4,7] Anai Gonzalez-Cordero,[1,5,7] and Robin R. Ali[1,2,4,*]

[1]UCL Institute of Ophthalmology, 11-43 Bath Street, London EC1V 9EL, UK
[2]Kellogg Eye Centre, University of Michigan, 1000 Wall St., Ann Arbor, MI 48105, USA
[3]Present address: Lorry I. Lokey Stem Cell Research Building, 265 Campus Drive, Stanford University, Palo Alto, CA, USA
[4]Present address: Centre for Cell and Gene Therapy, King's College London, Tower Wing, Guy's Hospital, London SE1 9RT, UK
[5]Present address: Children's Medical Research Institute, University of Sydney, 214 Hawkesbury Road, Westmead, NSW 2145, Australia
[6]These authors contributed equally
[7]These authors contributed equally
*Correspondence: robin.ali@kcl.ac.uk
https://doi.org/10.1016/j.celrep.2021.109022

SUMMARY

Age-related macular degeneration and other macular diseases result in the loss of light-sensing cone photoreceptors, causing irreversible sight impairment. Photoreceptor replacement may restore vision by transplanting healthy cells, which must form new synaptic connections with the recipient retina. Despite recent advances, convincing evidence of functional connectivity arising from transplanted human cone photoreceptors in advanced retinal degeneration is lacking. Here, we show restoration of visual function after transplantation of purified human pluripotent stem cell-derived cones into a mouse model of advanced degeneration. Transplanted human cones elaborate nascent outer segments and make putative synapses with recipient murine bipolar cells (BCs), which themselves undergo significant remodeling. Electrophysiological and behavioral assessments demonstrate restoration of surprisingly complex light-evoked retinal ganglion cell responses and improved light-evoked behaviors in treated animals. Stringent controls exclude alternative explanations, including material transfer and neuroprotection. These data provide crucial validation for photoreceptor replacement therapy and for the potential to rescue cone-mediated vision.

INTRODUCTION

Human sight is critically dependent upon the proper functioning of the macula, the cone-rich area of the retina responsible for high-acuity vision. Damage to the cone photoreceptors in this region, such as that caused by age-related macular degeneration (AMD) or inherited macular dystrophies like Stargardt's disease, can lead to devastating sight loss. The number of people in the UK alone affected by sight loss is set to reach 4 million by 2050 (Pezzullo et al., 2018). Present clinical approaches to treating photoreceptor degenerations are limited to slowing the progression of disease, leaving many patients with mid- to advanced-stage disease with no viable treatment options. Cell replacement therapy has the potential to reverse sight loss by replacing dead cells with healthy ones. Restoring functional connectivity following transplantation is an ambitious goal for CNS repair. However, the macula and cone-mediated vision represents one of the most feasible targets for this approach; it occupies a small, accessible area, and the connection of a relatively small number of new cones should yield useful vision.

A key requirement for the development of effective photoreceptor transplantation is the robust generation of bona fide photoreceptors. We and others have established protocols for generating large numbers of postmitotic pluripotent stem cell (PSC)-derived photoreceptors that can be used for transplantation (Gonzalez-Cordero et al., 2013, 2017; Kruczek et al., 2017; Meyer et al., 2009; Nakano et al., 2012; Zhong et al., 2014). Over the last decade, numerous preclinical studies have transplanted cell suspensions of (predominantly rod) photoreceptors derived either from neonatal mouse retina or mouse or human PSCs (hPSCs; see Aghaizu et al., 2017; West et al., 2020). In these studies, we and others have reported improvements in visual function in mouse models of stationary disease (Pearson et al., 2012) and progressive degeneration (Barber et al., 2013; Lamba et al., 2009; MacLaren et al., 2006; Singh et al., 2013; Zhu et al., 2017). Until recently, it was thought that the observed rescue was due to donor cells becoming incorporated within the

This is an open access article under the CC BY license (http://creativecommons.org/licenses/by/4.0/).

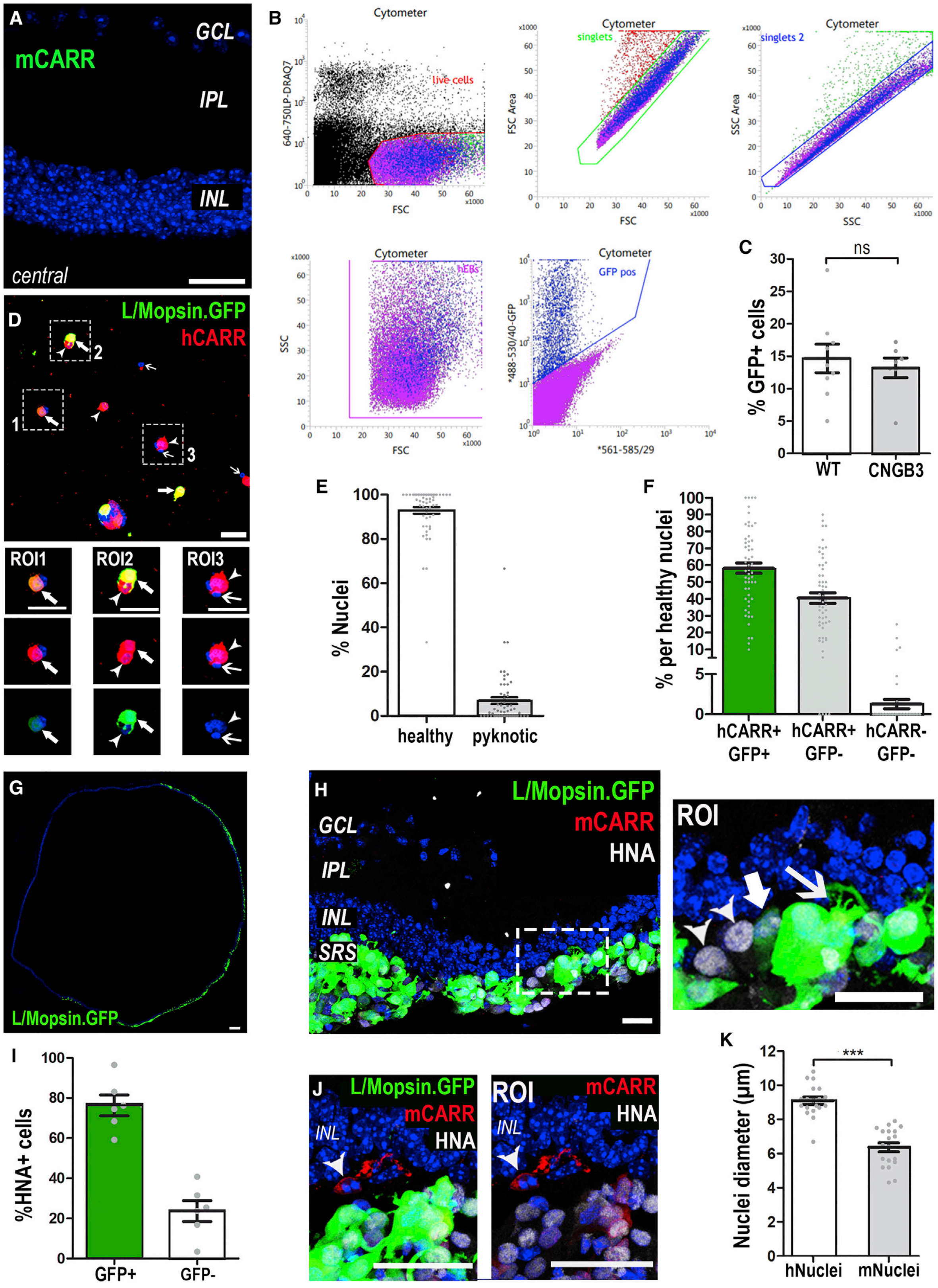

(legend on next page)

host retina. However, we and others have shown that where host photoreceptors remain, rescue is instead due to the transfer of cytoplasmic material between donor and host cells, which renders the host retina functional (Pearson et al., 2016; Santos-Ferreira et al., 2016; Singh et al., 2016). In light of these discoveries, a careful reinterpretation of the mechanisms underlying many previously reported rescues is required (see Nickerson et al., 2018).

There has since been renewed focus on the arguably more clinically relevant models of advanced degeneration, where the absence of most host photoreceptors largely, if not completely, removes confounding complications in interpretation that may arise from material transfer. To date, most reports of transplantation into advanced disease have used "sheets" of human fetal (see Seiler and Aramant, 2012) or rodent (Foik et al., 2018) retina, or "patches" cut from human (Iraha et al., 2018; Lin et al., 2020; McLelland et al., 2018; Shirai et al., 2016) and mouse (Assawachananont et al., 2014; Mandai et al., 2017) PSC-derived retinal organoids. These studies have yielded promising indications of improved function. However, rods make up the majority of the photoreceptors in these grafts, and the inclusion of inner retinal neurons, of unknown quantity and identities, within the grafted tissue limits the capacity for widespread donor photoreceptor-host bipolar cell (BC) contact. It is also not clear whether the reported improvements in predominantly rod-mediated function result from the formation of new donor-host synaptic connections or instead reflect activity arising within the graft itself, or a rescue of residual photoreceptor and/or other retinal neuronal function, due to neurotrophic effects or material transfer (see Reh, 2016). Indeed, an important limitation of many previous studies is the lack of appropriate controls for potential trophic and material transfer effects.

Transplantation of sorted photoreceptor cell suspensions represents an alternative approach that removes the barriers presented by inner retinal neurons included within the graft tissue. The majority of previous studies using this approach in advanced disease have transplanted rods isolated from neonatal retina or PSC-derived organoids (Barber et al., 2013; Barnea-Cramer et al., 2016; Singh et al., 2013). A recent report described the partial reprogramming of mouse fibroblasts into rod photoreceptors, which were transplanted into young *rd1* mice, leading to some improvements in visual function at low light levels (Mahato et al., 2020), although appropriate sham controls were lacking. Perhaps one of the most intriguing indications that transplanted human photoreceptors may make new connections comes from a recent study by Garita-Hernandez et al. (2019), who generated optogenetically engineered human induced PSC (hiPSC)-derived photoreceptors and recorded a modest improvement in retinal function in response to very bright light. Notably, however, the control was normal hiPSC-derived cones, which, in contrast with the data we present here, failed to yield any response.

Thus, to date, there is no conclusive evidence of transplanted human cone photoreceptors restoring cone-mediated visual function in advanced disease. Here, using histological and ultrastructural assessments and electrophysiology and behavioral tests, we show that transplanted purified hPSC-derived cones can make putative synaptic connections with host cells and improve photopic light-evoked retinal function and behaviors in the *rd1* mouse model of advanced retinal degeneration.

RESULTS

Generation of an immunodeficient retinal degeneration mouse model (*rd1/Foxn1^nu^*) and production of hPSC-derived functional and non-functional cones

To facilitate survival of transplanted human cells in the murine retina, we crossed the *rd1* model of rapid retinal degeneration (caused by a mutation in *PDE6β*) with the immunocompromised *Foxn1*^{nu} line. Characterization of *rd1/Foxn1*^{nu} mice at 3 months of age confirmed a complete loss of rods. Mouse Cone Arrestin^{+} (mCarr^{+}) cones were largely absent from the central retina (Figure 1A). A few remained toward the periphery that were of abnormal morphology, including an absence of inner (ISs) and

Figure 1. Isolation and transplantation of a pure population of human cones

(A) Three-month *rd1/FoxN1*^{nu} (*rd1*) central retina. Few, if any, mouse Cone Arrestin^{+} (mCarr; green) host cones were seen in the central retina. See Figure S1C for examples of cells in peripheral retina. n = 4 retinas.

(B) Representative FACS plot for sorting of L/Mopsin.GFP^{+} hESC-derived WT cones. Live, single GFP^{+} cells were sorted for purity. n = 16 FACS experiments.

(C) Viral infection with AAV.L/Mopsin.GFP targeting cone photoreceptors was equally efficient in both cell lines (mean ± SEM; n = 9 FACS experiments, WT line; n = 7 FACS experiments, CNGB3 line; unpaired t test).

(D) Viability and purity following FACS was assessed by plating and staining sorted cells for a human cone-specific marker, hCARR (red). hCARR^{+}/GFP^{+} (solid arrows, ROI1 and ROI2) and hCARR^{+}/GFP^{-ve} (arrowheads, ROI2 and ROI3) were observed. The few pyknotic cells were, without exception, hCARR^{-ve} (open arrows, ROI3).

(E) Quantification of cell viability of FAC-sorted cells, assessed by morphology of the nuclei (93% ± 2%, healthy nuclei; 7% ± 2%, pyknotic nuclei; n = 58 images, n = 2,047 cells) after plating.

(F) Quantification and verification of purity of the transplanted donor cell population. 99% of healthy cells were hCARR^{+} (59% ± 3% hCARR^{+}/GFP^{+} and 40% ± 3% hCARR^{+}/GFP^{-} versus 1% ± 1% hCARR^{-}/GFP^{-}; n = 58 images, n = 1,975 cells), despite variable levels of GFP expression.

(G) Montage image showing best spread of GFP^{+} cells, 12 weeks following transplantation of WT cones.

(H and I) L/Mopsin.GFP^{+} hESC-derived WT cones in the subretinal space (SRS) of 6-month-old *rd1* mice. GFP levels were very variable between cells (compare solid arow and open arrow). Almost all cells in the SRS expressed human nuclear antigen (HNA) (89% ± 7%), although a proportion of these had little detectable GFP (24% ± 5% HNA^{+}/GFP^{-ve}; mean ± SEM; n = 6 images, n = 6 animals; arrowheads). HNA^{+}/GFP^{+} cells (76% ± 5%) extended processes toward the host retina (arrows).

(J) Host mCARR^{+} cones (red; arrowhead) did not express HNA.

(K) HNA^{-ve} host and HNA^{+ve} human donor cones exhibited a significant difference in nuclear size (6 ± 1 μm versus 9 ± 1 μm, respectively; p < 0.001, Mann-Whitney test; mean ± SD, n > 3 retinas, n = 20 nuclei).

Scale bars, 25 μm (A, D, H, and J); 100 μm (G). GCL, ganglion cell layer; INL, inner nuclear layer; IPL, inner plexiform layer.

outer segments (OSs) and/or axonal processes (Figures S1A–S1C). Gross organization, morphology, and remodeling of the interneurons were consistent with previous reports for similarly aged *rd1* mice (Lin et al., 2009; Zhang et al., 2009) (Figures S1D–S1G).

We developed a hiPSC line to generate cone photoreceptors incapable of light-mediated function, to use as a stringent control for our analysis of functional connectivity. The hiPSC line (Figures S2A–S2D) was generated from a patient with achromatopsia due to a homozygous deletion (c.1148delC) in *CNGB3,* encoding the beta subunit of a cone-specific cyclic nucleotide-gated (CNG) channel. The mutation results in truncated CNGB3 polypeptides leading to impaired CNG channel function and phototransduction (Peng et al., 2003). Patients display complete achromatopsia (no cone function), although the cones themselves do not die until relatively late in disease (Dubis et al., 2014; Hirji et al., 2018; Liu et al., 2013). Retinal organoids were generated from a normal, healthy human embryonic stem cell (ESC) line (herein called "WT") and from the CNGB3 iPSC line (herein called "CNGB3"), using our previously established protocol (Gonzalez-Cordero et al., 2017). The organoids from each line were morphologically similar (Figures S3A and S3B) with no notable differences between WT and CNGB3 cones following immunohistochemical characterization throughout the culture period (Figures S3C–S3F).

Survival of purified hPSC-derived cones in the *rd1/Foxn1^nu* retina

At week 13 of differentiation, cone photoreceptors were labeled using adeno-associated virus (AAV)-ShH10(Y445F)-containing GFP under the control of an L/Mopsin promoter (L/Mopsin.GFP), which specifically labels human cones (Gonzalez-Cordero et al., 2017, 2018). At week 17, large numbers of GFP$^+$ cells displaying typical cone morphology were observed in both WT and CNGB3 organoids. Organoids were dissociated, and GFP$^+$ cones were isolated by fluorescence-activated cell sorting (FACS), selecting for purity (Figure 1B); a similar percentage of total cells were GFP$^+$ for both lines (Figure 1C). Their identify as a pure population of viable cones was further verified by immunostaining with human-specific CONE ARRESTIN (hCARR); 98% ± 3% of healthy sorted cells were hCARR$^+$ (Figures 1D–1F). Following isolation, 500,000 GFP$^+$ cone photoreceptors/eye were transplanted into the subretinal space in the superior central retina of 3-month-old *rd1/Foxn1^{nu}* mice, and animals were assessed 3 months post-transplantation, unless otherwise stated. Approximately 70% of WT cone transplants (n = 32 injected eyes) and 90% of CNGB3 cone transplants (n = 23) were successful (see STAR Methods for definition). Fundoscopy showed a comparable range of cell mass size from both cell lines (Figures S3G and 3H). In some eyes, the cell mass occupied a third of the retina (Figure 1G), while in other eyes it was more focal. Significant cell masses were observed at least 4 months post-transplantation (longest time point examined).

Immunostaining for human nuclear antigen (HNA) and mCarr further confirmed the human origin of the subretinal cell mass. Almost all cells within the subretinal space labeled for HNA (89% ± 7%; n = 11 eyes; Figure 1H) and all GFP$^+$ cells were positive for HNA. Very similar results were found in eyes that received CNGB3 cones (Figures S4A–S4C; n = 14 eyes). Note that although all sorted cells are GFP$^+$ at the time of transplantation (Figure 1B), the level of GFP within any given cell can vary significantly because of viral expression levels (Figure 1H, ROI). As such, some appear to show little/no GFP compared with very high GFP in neighboring cells, as detected by imaging (24% ± 5% of HNA$^+$ cells; Figure 1I). The vast majority of HNA$^+$ cells were also negative for mCarr (Figures 1H and 1J), with minimal cross staining (~3% of HNA$^+$ cells; Figure 1J, ROI). Occasional mCarr$^+$/HNA^{-ve} host cones were seen, but these did not co-label with either GFP (Figure 1J) or hCARR (Figure 2B, ROI2). The nuclei of mCarr$^+$/HNA^{-ve} host mouse cones were significantly smaller than HNA$^+$ transplanted human cones (Figure 1K; 6 ± 1 μm versus 9 ± 1 μm, respectively; mean ± SD, $p < 0.001$; Mann-Whitney test; n = 20 nuclei; n > 3 retinas).

Purified hESC-derived cones mature and form nascent OSs following transplantation

The cell masses observed here were much larger than previous transplants of (smaller numbers of) hPSC-derived cones (Gonzalez-Cordero et al., 2017). In those previous studies, maturation of the transplanted cells was incomplete, with very few transplanted cells expressing late maturation markers, such as Mopsin and Peripherin-2. We considered that the increased density and spread of the cell mass achieved here may assist in the maturation of transplanted dissociated cones. Almost all transplanted cells expressed hCARR (Figure 2B; n = 10 eyes), as expected from the pre-transplantation assessments (Figures 1D and 1F). Moreover, ~80% also expressed Mopsin (Figures 2A and 2B; N.B. this antibody also labels hCARR^{-ve} mouse cones; see Figure 2A, ROI2). Although much of the labeling for Mopsin is cytoplasmic, ~20% of human WT cones presented nascent segment-like protrusions (Figure 2A), many projecting toward the RPE (retinal pigment epithelium) (Figure 2B, ROI1). Consistent with these being nascent OSs, ~25% of transplanted cells displayed elongated PRPH2$^+$ structures (Figures 2C and 2E) that were typically located adjacent to mitochondria-rich regions, typical of ISs (Figure 2D). CNGB3 cones showed similar features (Figure S4D). We further validated this by transmission electronic microscopy (TEM); this is technically challenging due to the relative disorganization of transplanted cells, but we observed many regions containing structures resembling ISs, packed with mitochondria, which were often adjacent to small stacks of membranous disks, typical of OS morphology (Figures 2F and 2G). These OS-like structures were more abundant in areas of denser cell mass and were never found outside the area of transplantation.

Host BPs demonstrate dendritic remodeling after transplantation of hPSC-derived cones

We next assessed the host retinal response to the presence of large numbers of human cones (observations based on n = 10 *rd1/Foxn1^{nu}* + WT cones; n = 8 eyes *rd1/Foxn1^{nu}* + CNGB3 cones). We first assessed glial scaring, a common remodeling event that could impede donor-host interactions (Barber et al., 2013; Strettoi, 2015). Widespread GFAP upregulation in host Müller glia cells was evident, but no glial scar was apparent; instead GFAP$^+$ processes extended into the cell mass,

CellPress
OPEN ACCESS

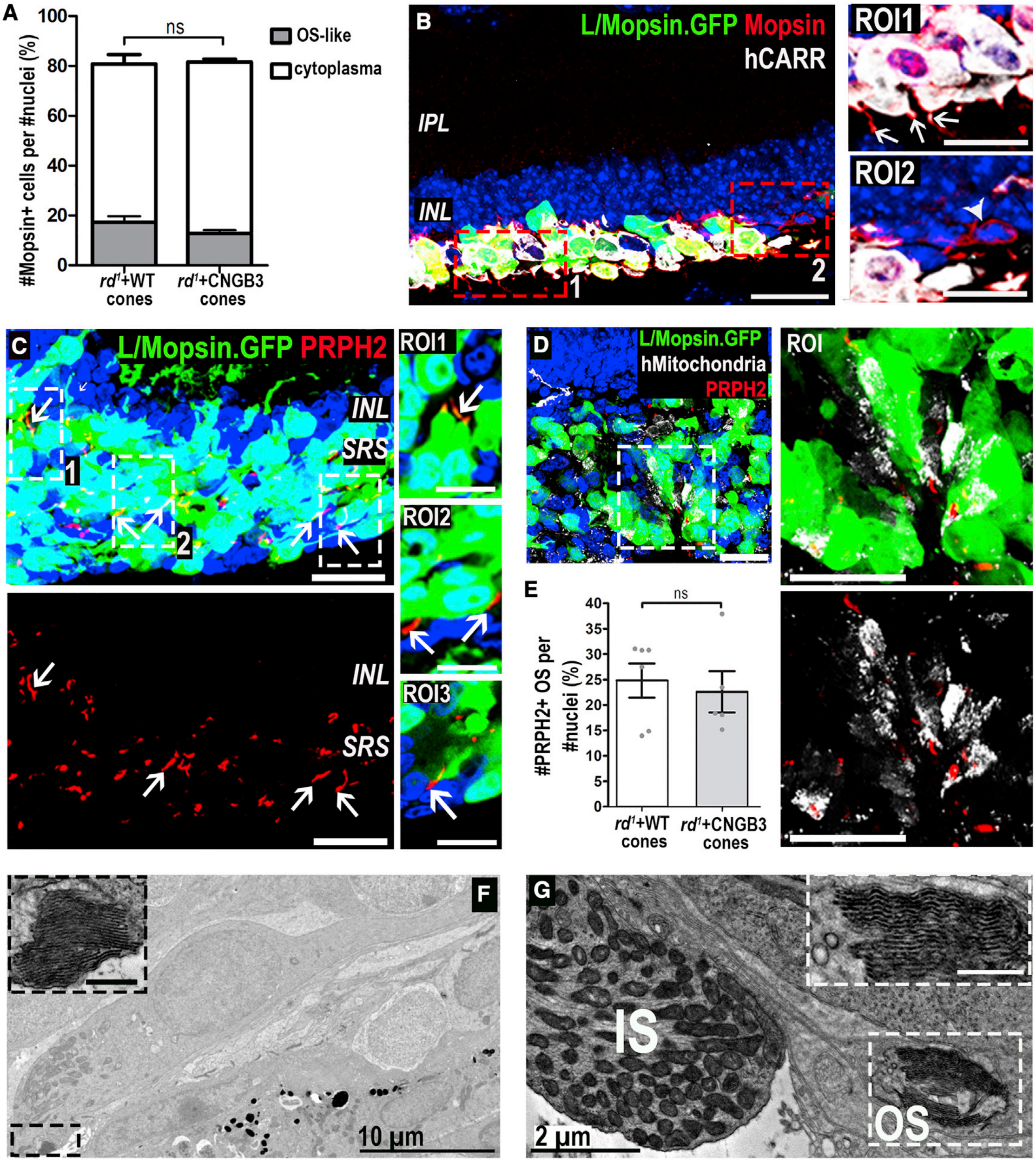

Figure 2. Maturation of human WT cones following transplantation as a purified cell suspension into the *rd1/FoxN1^{nu}* model of advanced degeneration

(A) Quantification of percentage of cells in SRS that were MOPSIN^{+} and formed segment-like structures as a proportion of HNA^{+} nuclei found within the cell mass (81% ± 6% MOPSIN^{+} cells versus 82% ± 2%, WT versus CNGB3, no significant [N.S.] differences between WT versus CNGB3, Mann-Whitney test; mean ± SEM). A proportion of these cells displayed MOPSIN localized to nascent segment-like structures (17% ± 3% versus 13% ± 1%, WT versus CNGB3; N.S., Mann-Whitney test).

(B) GFP^{+} cells (green) expressing hCARR (white) and MOPSIN (red), which localized to nascent segment-like structures in some cells (ROI1, arrows). Rare host M-cones were identified, but no segment-like structures were associated with these cells (ROI2, arrowhead).

(C) Numerous PRPH2^{+} (red) segment-like structures (arrows) can be seen within the cell mass. ROI1–3, single confocal sections through some segment-like structures.

(D) Human (h) mitochondria-rich (white) structures were in close proximity to PRPH^{+} structures (ROI and dual channel).

(E) Quantification of number of PRPH2^{+} segment-like structures as proportion of nuclei found within the cell mass (25% ± 3% versus 23% ± 4%, WT versus CNGB3; N.S., Mann-Whitney test; mean ± SEM, n > 5 images, n > 5 retinas per group).

(F and G) Representative TEM images of structures consistent with mitochondria-rich ISs and stacked disks of OSs (ROI).

Scale bars, 25 µm (A–D); 12.5 µm (ROIs in B and D); 0.5 µm (ROI in F and G). IS, inner segment; OS, outer segment.

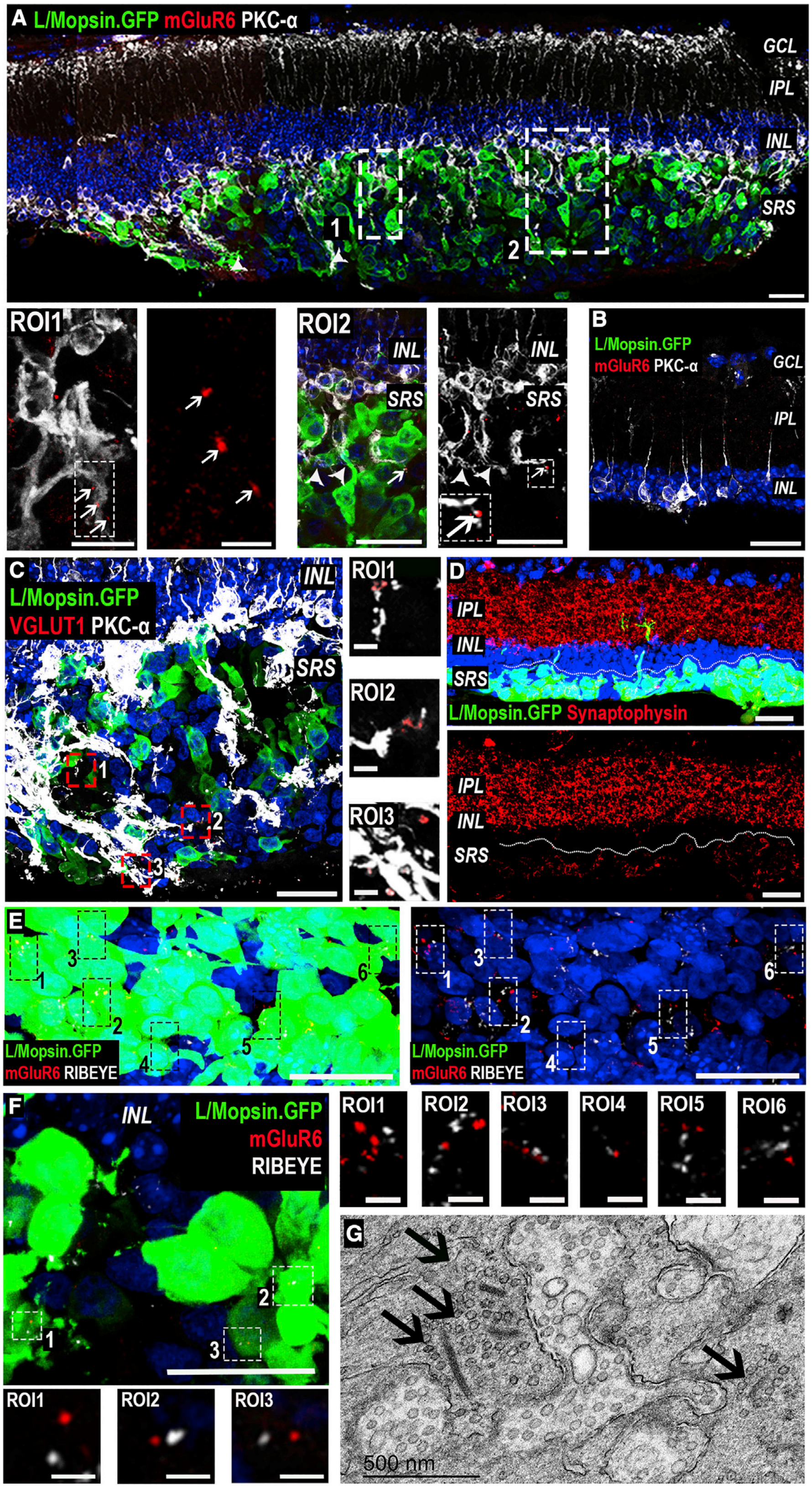

(legend on next page)

appearing to wrap around the transplanted cones (Figures S4F and S4G).

We then studied the interaction between host BCs and transplanted human cones. The retraction of BC dendrites is a typical remodeling event following photoreceptor degeneration (D'Orazi et al., 2014; Jones and Marc, 2005; Strettoi, 2015). Although it was not possible to efficiently label host cone BCs, as a corollary we observed host protein kinase C (PKC)-α-positive rod BCs extending dendrites into the cell mass after transplantation. This was seen both in those eyes receiving WT cones (Figure 3A) and those receiving CNGB3 cones. No host BC dendrites extended into the subretinal space in the absence of transplanted cells (Figure 3B).

Both rod and cone ON BCs express the post-synaptic metabotropic glutamate receptor 6 (mGluR6) in their dendritic synaptic terminals, and this expression is lost in degeneration (D'Orazi et al., 2014; Puthussery et al., 2009). We confirmed that mGluR6 protein was absent from BC dendrites in untreated *rd1/Foxn1^{nu}* mice (Figure 3B; Figures S1D and S1E). Encouragingly, we saw at least partial re-expression of mGluR6, often in PKC-α positive dendrites, around the site of transplantation in both WT and CNGB3-transplanted eyes. Quantitatively, this yielded an 8.7-fold (±3.7 SD; integrated density measurements) increase in mGluR6 expression in the region of transplantation compared with the same retina away from the site of transplantation (n = 5 eyes).

Collectively, these data indicate a remarkable degree of plasticity and remodeling of the host retinal synaptic circuitry in response to the presence of transplanted human cones. These changes were restricted to areas where a dense cell mass was present, suggesting transplanted cones may alter their local microenvironment.

Transplanted human cone photoreceptors make synaptic contacts with host BPs

The upregulation of mGluR6 expression by host BCs indicated the potential for *de novo* synaptogenesis between transplanted human cones and host interneurons, which we sought to characterize further (n > 4 eyes/group). Vesicular glutamate transporter 1 (VGlut1), a marker of pre-synaptic glutamate vesicles, was readily detectable throughout all cell masses examined (Figure 3C). We next confirmed the presence of the presynaptic proteins Synaptophysin (Figure 3D) and RIBEYE (Figures 3E and 3F) throughout the transplanted cell mass. RIBEYE protein is essential for the formation of synaptic ribbons and, in areas where the cell mass was denser, was often seen in close proximity to mGluR6 puncta, as putative RIBEYE/mGluR6 "synaptic complexes" (Figures 3E, 3F, and S4E). Qualitatively, regions containing sparser transplanted cones showed less frequent staining for both markers and fewer complexes. TEM analysis revealed the presence of ribbon synapses within the cell mass (Figure 3G). In some cases, multiple ribbons were visible within the same pedicle, consistent with cone synapse morphology. No ribbons were detected outside the injection area or in any untreated control. Together, these data provide strong evidence that transplanted human cone photoreceptors can establish putative synaptic connections with the host retina.

Transplantation of purified human WT cones restores light-evoked mERGs and spiking activity in the host retina

To determine whether transplanted human WT cones can drive downstream light responses in host retinal ganglion cells (RGCs), thereby restoring photosensitivity to the degenerated murine retina, we recorded microelectroretinograms (mERGs) and spiking activity from host RGCs using multielectrode array (MEA) recordings of explanted retinas (Figure 4; Table S1). Untreated *rd1/Foxn1^{nu}* retinas possessed oscillatory local field potentials (LFPs), confirming strong contact between retina and electrodes, but did not show any light-evoked mERGs (Figure 4A, left; Table S1). Similarly, the peristimulus time histogram (PSTH) of spiking activity showed no response in the vast majority of channels (Figure 4A, right). The *rd1* mouse is often (incorrectly) described as being non-light responsive; this is an oversimplification, because several groups have described the presence of slow, low-amplitude increases in firing rate following light onset. These arise from deafferented intrinsically photosensitive RGCs (ipRGCs) that express melanopsin (Davis et al., 2015; Provencio et al., 2000; Schroeder et al., 2018). These responses can be observed in isolation only when photoreceptor input is absent, as in advanced degeneration (Brown et al., 2010; Eleftheriou et al., 2020; Procyk et al., 2015) or under pharmacological intervention (Wong, 2012; Wong et al., 2007). Accordingly, we observed a small proportion of channels presenting similar slow, low-amplitude increases in firing rate following light onset, which recovered slowly after light offset (channel with asterisk, Figure 4A).

We used *Gnat1$^{-/-}$* retinas as a positive control for cone-mediated function. These mice lack rod α-transducin rendering rods non-functional but viable, and all photoreceptor-driven light responses are thus cone derived (Calvert et al., 2000). In contrast with untreated *rd1* mice, *Gnat1$^{-/-}$* retinas exhibited widespread light-evoked mERGs in response to photopic stimuli (Figure 4B; Table S1), which were time locked to stimulus onset and offset.

Figure 3. Remodeling of host *rd^1* retina and formation of putative synaptic-like connections

(A) Large numbers of GFP$^+$ WT cones in close proximity to the host inner retina. Host *rd1/FoxN1^{nu}* (*rd^1*) PKC-α^+ rod bipolar cells (BCs) extended processes into the cell mass (ROI1, ROI2, arrowheads). Post-synaptic protein, mGluR6, was seen within those processes (ROI1, ROI2, arrows).

(B) BCs do not extend dendrites beyond the inner retina, and mGluR6 is not expressed in untreated eyes.

(C) Vesicular glutamate transporter 1 (VGLUT1) was seen in the cell mass in close proximity to host *rd^1* BC dendrites (ROI1–3).

(D) Pre-synaptic protein Synaptophysin was present in the host IPL and in transplanted GFP$^+$ cones.

(E and F) In areas of high donor cell density, numerous putative RIBEYE/mGluR6 complexes were seen in close proximity to one another (ROIs). Images are representative of n = 10 WT and n = 8 CNGB3-transplanted eyes.

(G) TEM image showing presence of ribbon synapses (arrows), including multiple ribbons within a single terminal (left), within the area of injection.

Scale bars, 25 μm (A–C and E); 12.5 μm (ROI2 in A and F); 3 μm (ROI1 in A, ROI3 in C and F); 500 nm (G).

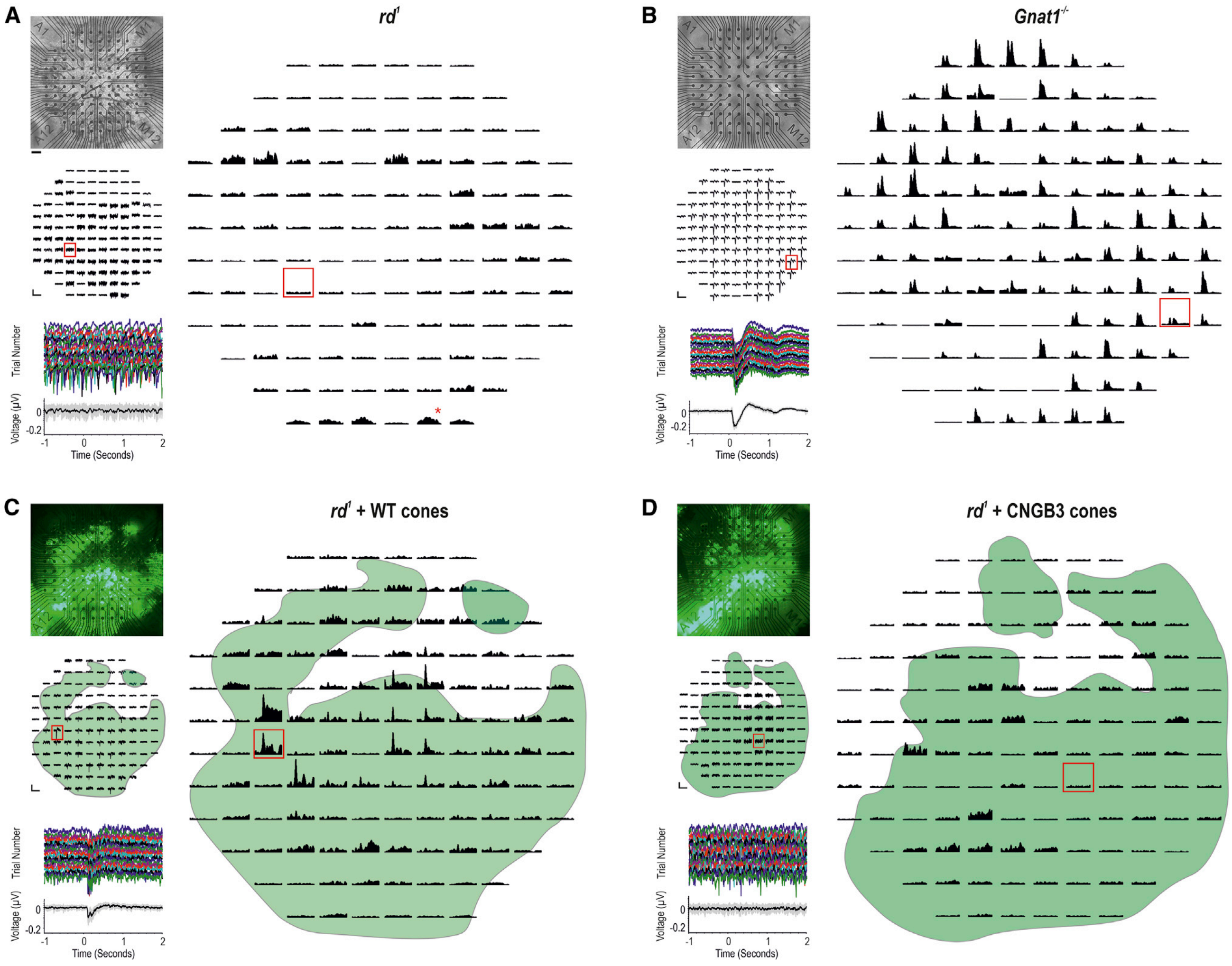

Figure 4. Transplantation of human WT cones generates widespread mERGs and light-evoked spiking activity in the host rd^1 retina

(A, top left) Representative untreated *rd1/FoxN1*^{nu} (rd^1) retina on MEA. (Middle left) There were no discernible light-evoked mERGs from the same retina. (Bottom left) Magnified trace from a representative channel (red box) shows individual trials and mean (±SD) (bottom). (Right) PSTH (mean ± SEM) of multi-unit spiking. The majority of channels are non-light responsive. A few channels show slow, sustained, deafferented ipRGC responses following light onset (red asterisk).

(B, top left) Representative *Gnat1*$^{-/-}$ retina. (Middle left) Most channels exhibit light-evoked mERGs. (Bottom left) Magnified trace (red box) shows reproducibility of mERG across individual trials and mean (±SD) (bottom). (Right) The same channels show transient, large-amplitude changes in firing rate at light onset and/or offset.

(C, top left) Representative rd^1 + WT cone-transplanted retina. (Middle left) mERGs are present on a large proportion of channels and correlate with position of GFP$^+$ cell mass (green overlay). (Bottom left) Magnified trace (red box) shows reproducibility of the response. (Right) A large proportion of channels within cell mass show transient, large-amplitude increases in firing rate at light onset and/or offset.

(D, top left) Representative rd^1 + CNGB3 cone-transplanted retina. (Middle left) No discernible mERGs were seen. (Bottom left) Magnified trace (red box) demonstrates no light-evoked mERGs. (Right) Most channels are not light responsive; a few channels demonstrated deafferented ipRGC responses following light onset.

Scale bars: 100 µV, 3 s (middle panels, A, C, and D); 250 µV, 3 s (middle panels, B); 100 spikes/s, 5 s (right panels, A, C, and D); and 200 spikes/s, 5 s (right panel, B). In (C) and (D), green represents GFP^{+ve} WT cones and GFP^{+ve} CNGB3 cones, respectively. See Table S1 for n values.

Multi-unit spiking activity showed fast, large-amplitude, cone-driven changes in firing rate (Figure 4B, right) readily distinguishable from the residual deafferented ipRGC responses seen in untreated *rd1* retinas (Figure 4A).

Following transplantation, we consistently observed light-evoked mERGs in *rd1/Foxn1*^{nu} retinas receiving human WT cones (Figure 4C; Table S1). These were reproducible and time-locked to stimulus onset. The GFP$^+$ cell mass encompassed 70% (±2; n = 6) of total area, and its position strongly correlated with the presence of mERGs (Figure 4C, left). We further examined the multi-unit spiking activity within the GFP-expressing region (Figure 4C, right) and found that a large proportion of channels exhibited fast, transient light-evoked changes in firing rate that correlated with the presence of mERGs and were not observed on channels beyond the spatial extent of the cell mass.

Finally, to exclude material transfer and neuroprotection as mechanisms of re-photosensitizing the degenerate retina, we examined *rd1/Foxn1^{nu}* retinas that had received CNGB3 cones (Figure 4D). This control is identical to WT cones in every way other than the absence of the CNGB3 channel. It thus has the same potential to provide neurotrophic support and to transfer the same profile of molecules as normal cones, with the exception of the CNGB3 channel. As for untreated *rd1/Foxn1^{nu}* retinas, oscillatory LFPs were observed, but there were no discernible light-evoked mERGs following stimulus presentation (Figure 4D, left; Table S1). There was no qualitative difference between LFPs inside and outside the regions of GFP expression, and the majority of channels were not light responsive (Figure 4D, right); a small proportion of channels presented a response profile that is typical of those reported for deafferented ipRGCs. Together, these confirm the lack of functional output from CNGB3 cones and the absence of rescue of any remaining host cones by material transfer or any change in host RGC function through other neuroprotective means.

Transplanted human WT cones communicate with host BPs by glutamatergic transmission

To confirm that the light-evoked responses recorded in WT cone-transplanted retinas originate from the transplanted cones and are mediated via glutamatergic transmission, we repeated the light stimulus protocol before, during, and after the addition of synaptic blockers (L-AP4, DNQX, and D-AP5). Together these drugs block all known metabotropic and ionotropic glutamatergic transmission of visual information at the photoreceptor-BC synapse. As expected from the literature (Wong et al., 2007; Zhao et al., 2014), the slow, sustained responses seen in untreated *rd1/Foxn1^{nu}* were not eradicated by synaptic blockers and remained after washout, confirming their origin from deafferented ipRGCs and not from any residual host cones and BCs (Figure 5A). By contrast, in *Gnat1$^{-/-}$* retinas, addition of synaptic blockers reversibly eradicated all fast, transient responses, confirming they originated from glutamatergic transmission between cones and BCs in the outer retina (Figure 5B). Similarly, the widespread and fast light-evoked spiking activity present in *rd1/Foxn1^{nu}* transplanted with human WT cones was reversibly inhibited by glutamatergic synaptic blockers (Figure 5C). This provides strong evidence that these responses originate from transplanted cones and are transmitted via a glutamatergic synapse between human cones and murine host BCs. Note that in both *Gnat1$^{-/-}$* and transplanted *rd1/Foxn1^{nu}* retinas, occasional deafferented ipRGC responses were detected but only under pharmacological synaptic blockade (channels with asterisks, Figures 5B and 5C) and are analogous to physiological conditions in the untreated *rd1/Foxn1^{nu}* retina (Wong et al., 2007; Zhao et al., 2014). In CNGB3 cone-transplanted retinas (Figure 5D), the majority of channels demonstrated no response to light, with occasional deafferented ipRGC-like responses that were unaffected by glutamatergic synaptic blockade.

Transplanted human WT cones drive a diverse array of fast and large-amplitude visual responses at behaviorally relevant light levels

We next spike-sorted the data to quantify the response characteristics of individual host RGCs (Figure S5; Figure 6). WT cone-transplanted *rd1/Foxn1^{nu}* retinas elicited a significantly higher proportion of light-responsive units than either untreated or CNGB3 cone-transplanted *rd1/Foxn1^{nu}* retinas (52% ± 9% versus 0.01% ± 0.01% or 4% ± 3%, respectively; Figure 6A). In *Gnat1$^{-/-}$* mice, single units could be categorized into 10 quantitatively distinct response profiles based on their response to the 1-s light stimulus (Table S2; see STAR Methods for automated single-unit response classification; N.B. these do not necessarily correspond to a particular class of RGC). The average PSTH profiles for each of the 10 groups for each experimental intervention are shown in Figure 6B. In *rd1/Foxn1^{nu}* retinas receiving WT cones, we could identify all of these light-response types, with the exception of "Suppressed ON-OFF" type responses (Figure 6C). This is in striking contrast with the small number of light-responsive units from both untreated *rd1/Foxn1^{nu}* and those that received CNGB3 cones, both of which exhibited response profiles consistent only with deafferented ipRGC signaling (Figure 6C). Qualitatively, the range of response types in WT cone-transplanted *rd1/Foxn1^{nu}* retinas was similar to that of *Gnat1$^{-/-}$*, although there was a prevalence of ON-type responses (Figure 6D; 52% versus 11% of light-responsive units, respectively) and a reduction in ON-OFF-type responses (13% versus 50%) (Figure S6A). The variety of response profiles could be observed in all retinas assessed (Figure S6B). This is important because it means that transplanted WT cones must be relaying visual information to both ON and/or OFF BCs in order to drive the parallel processing pathways of the inner retina from which this variety of responses originates. Moreover, the complexity of the responses recorded also suggests that visual processing conducted by other host interneurons, such as amacrine cells, remains at least partially intact.

We next examined response amplitude and latency. Responses were grouped into ON (units that increased firing rate at light onset) and OFF (units that increased firing rate at light offset). In WT cone-treated retinas, response amplitudes for the ON (Figure 6E) and OFF (Figure 6F) components were significantly smaller compared with *Gnat1$^{-/-}$* controls. In contrast, the latency-to-peak response for the ON component in WT cone-transplanted *rd1/Foxn1^{nu}* was as fast as those in *Gnat1$^{-/-}$* mice (Figure 6G; $p = 0.76$; unpaired t test), whereas the latency for the OFF component was significantly slower (Figure 6H). Many factors affect the amplitude and latency of light-evoked RGC responses derived from transplanted cones, including OS length, phototransduction efficiency, synaptic transmission, the physiology of interneurons in the degenerate retina, or a combination of any/all of these. Nonetheless, the values recorded here for WT cone-transplanted *rd1/Foxn1^{nu}* retinas are within the expected parameters required for behaviorally relevant cone-based vision.

Finally, we examined retinal responses at different light intensities. The average PSTHs at each irradiance are shown in Figure 6I. The average change in firing rate demonstrated that WT cone-transplanted *rd1/Foxn1^{nu}* retinas show a sensitivity to light intensity that is similar to *Gnat1$^{-/-}$* (Figures 6I and 6J; $p > 0.999$, the exception being response to 10^{14} photons, where $p = 0.101$; two-way ANOVA with Bonferroni correction). Thus, the transplanted human WT cones are functioning within the normal cone photoreceptor sensitivity range and at natural daylight levels.

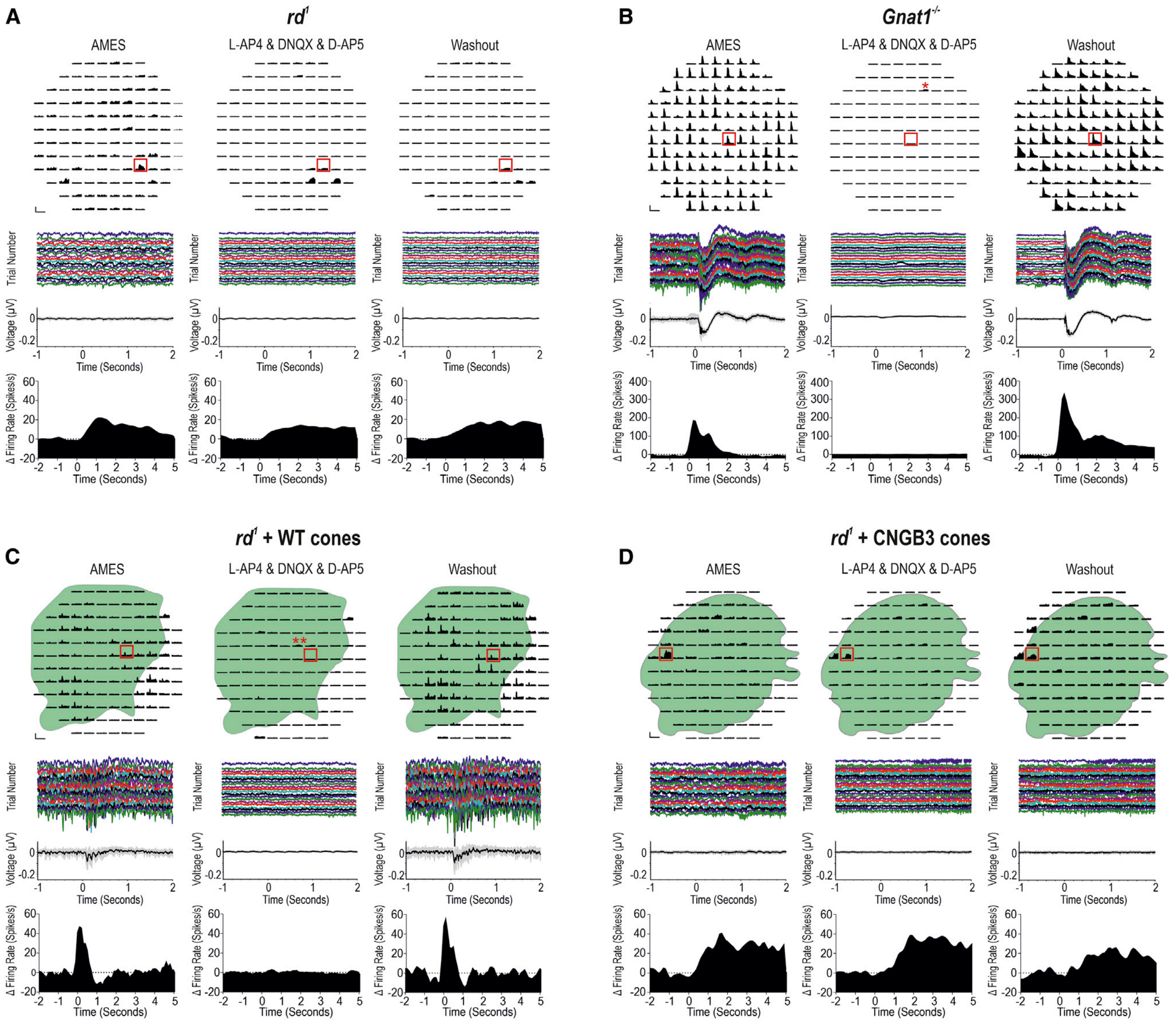

Figure 5. Transplanted human WT cones connect to the host rd^1 retina through functional glutamatergic synapses in the outer retina
Multi-unit spiking activity before, during, and after synaptic blockade.
(A) Untreated rd^1 retina. Most channels were not light responsive. No discernible mERGs were seen. A few channels demonstrated deafferented ipRGC responses following light onset (red box), which were not eradicated by synaptic blockers, as expected (Wong et al., 2007).
(B) $Gnat1^{-/-}$ retina. Transient increases and decreases in firing rate were observed following light onset and/or offset. These were reversibly abolished by synaptic blockers (asterisk [*] denotes deafferented ipRGC responses observed only with pharmacological intervention). mERG and multi-unit spiking activity (red box) shows fast cone-driven response, which is reversibly abolished by pharmacological intervention.
(C) Rd^1 + WT cone-transplanted retina. Transient increases in firing rate correlated with the position of overlying GFP^{+ve} WT cones. Addition of synaptic blockers reversibly abolished these responses (double asterisks [**] denote deafferented ipRGC response visible under synaptic blockade). Light-evoked mERG and multi-unit spiking activity (red box) illustrates WT cone-driven light responses that are both abolished by pharmacological intervention and return following washout.
(D) Rd^1 + CNGB3 cone-transplanted retina. Most channels were not light responsive. A few channels show a slow, sustained increase in firing rate following light onset (red box) that was unaffected by synaptic blockers, indicating that these responses originate from deafferented ipRGCs.
Scale bars: 100 spikes/s, 5 s (A, C, and D); 200 spikes/s, 5 s (B). See Table S1 for n values.

Transplanted human WT cones rescue visual function in advanced degeneration

We next sought to determine whether the restoration of retinal function shown above is sufficient to rescue visually evoked behaviors. The *rd1/FoxN1^{nu}* is unsuitable for traditional visual assessments like water-maze and optomotor testing due to rapid degeneration preventing pre-treatment training and an inherent head drift. Instead, we used a light avoidance assay because mice are nocturnal and, if able to detect light, will avoid bright open spaces. Mice were placed in the light compartment

CellPress
OPEN ACCESS

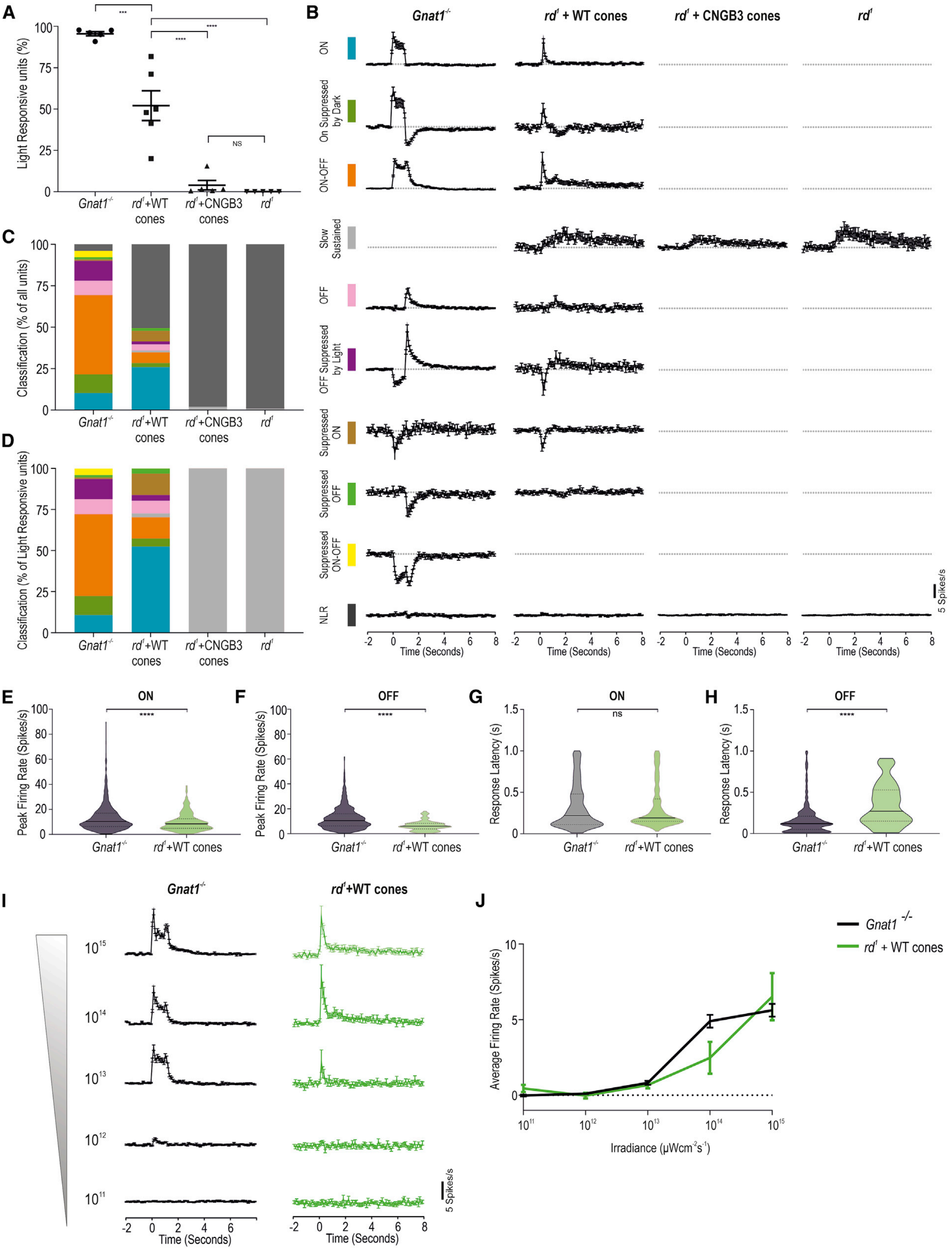

(legend on next page)

of the arena, close to the aperture between light and dark compartments (Figures 7A and 7B), and allowed to roam. Wild-type control mice showed a clear preference for the dark compartment, while untreated *rd1/Foxn1^{nu}* showed no such preference (82% total time ± 7% versus 34% ± 7%; n = 10 versus n = 17 mice, respectively; mean ± SEM). *Rd1/Foxn1^{nu}* mice receiving CNGB3 cone transplants also showed no preference (43% ± 7%; n = 15 mice). In contrast, *rd1/Foxn1^{nu}* mice that received WT cones showed a clear and significant preference for the dark compartment (68% ± 7%; n = 17 mice), and the time spent in the dark by these animals was not significantly different from wild-type mice (Figure 7C).

The time taken to first cross to the dark compartment provides a quantitative measure of light aversion (Figure 7D). In comparison with wild-type animals (0.02 ± 0.01 min), untreated *rd1/Foxn1^{nu}* mice (3.7 ± 1.0 min) and mice that received CNGB3 cones (3.2 ± 1.0 min) took significantly longer to cross. In contrast, animals that received human WT cones showed a shorter average latency (1.2 ± 2.0 min) than both untreated and CNGB3 transplant controls, which was slower but statistically similar to wild-type animals. Representative tracking plots from individual animals (Figures 7E–7H; Figure S7) and heatmap plots showing the average time spent in any given arena location (Figures 7I–7L) show that WT cone-transplanted *rd1/Foxn1^{nu}* animals displayed behaviors most similar to that of wild-type mice (Figures 7K and 7L) and markedly different from those of untreated or CNGB3-transplanted *rd1/Foxn1^{nu}* controls.

DISCUSSION

Photoreceptor replacement therapy offers significant hope as a treatment for advanced retinal degenerations (West et al., 2020). Of particular importance is the ability to transplant human cone photoreceptors to treat the macular, the region we are most dependent upon for cone-mediated, high-acuity vision. Here, we provide definitive evidence of rescue of both retinal and visual function following the transplantation of a purified population of hPSC-derived cone photoreceptors into a mouse model of advanced retinal degeneration. We show that human cones transplanted as a purified cell suspension can mature *in vivo* to form nascent light-sensing OSs, pre-synaptic ribbons, and putative synapses. We report a surprising degree of complexity in the type of light-evoked host retinal response, indicating significant inner retinal plasticity and processing. Moreover, this activity is sufficient to drive light-evoked behavior. Crucially, sham transplants of non-functional CNGB3 hiPSC-derived cone photoreceptors yielded no functional rescue.

Although there are reports of improvements in retinal and/or visual function following transplantation into advanced disease, the majority have used donor-derived or PSC-derived retinal sheets or dissociated cells, each containing a mixed population of retinal progenitors and/or rod photoreceptors (Barnea-Cramer et al., 2016; Garita-Hernandez et al., 2019; Iraha et al., 2018; Mandai et al., 2017; McLelland et al., 2018; Shirai et al., 2016). The transplantation of pre-organized retinal sheets is an attractive approach for advanced disease, and such transplants can exhibit good maturation within the rosettes formed by the grafted tissue. However, the efficacy of this approach is significantly impaired by the presence of donor interneurons and glia, which reduce contact and connectivity between donor (mostly rod) photoreceptors and host BCs (Assawachananont et al., 2014; Iraha et al., 2018; Mandai et al., 2017). This impediment is removed with the transplantation of a purified population of cone photoreceptors, as used here. Furthermore, although improvement in rod-mediated vision may be worthwhile, rescuing cone-mediated vision represents a far greater benefit to patients. This is, to our knowledge, the first conclusive demonstration of restoration of photopic visual function in advanced disease following the transplantation of healthy human cone photoreceptors.

Of particular significance in the current study is the inclusion of appropriate sham controls. Cone photoreceptors derived from achromat CNGB3 (c.1148delC) hiPSCs are non-functional but otherwise similar to healthy hESC-derived cones, for at least the duration of this study (Dubis et al., 2014; Hirji et al., 2018; Liu et al., 2013). The use of this control is vital. First, it controls for functional rescue by material transfer, the intercellular exchange of cytoplasmic material between donor and host photoreceptors that can rescue remaining host photoreceptors (Pearson et al., 2016; Santos-Ferreira et al., 2016; Singh et al., 2016). The CNGB3 cells contain the same array of potentially transferrable molecules as WT cones, with the exception of

Figure 6. Transplanted human WT cones drive an array of fast, large-amplitude visual responses at physiological light levels

(A) Percentage of light-responsive units in *rd^{1}* + WT cone-transplanted retina (n = 6) was lower than *Gnat1$^{-/-}$* (n = 5) but significantly higher than those in untreated *rd^{1}* retinas (p < 0.0001; one-way ANOVA) and *rd^{1}* + CNGB3 cone-transplanted retina (p < 0.0001, one-way ANOVA). N.S. difference between untreated *rd^{1}* and *rd^{1}* + CNGB3 cone (p = 0.96, one-way ANOVA).

(B) Average PSTH of single units categorized into 10 quantitatively defined types based on their response to a 1-s light step in *Gnat1$^{-/-}$* (n = 1,031 units), *rd^{1}* + WT cone (n = 687 units), *rd^{1}* + CNGB3 cone (n = 945 units), and untreated *rd^{1}* (n = 641 units). Time bin = 0.1 s; lights on at time = 0.

(C) Quantification of the distribution of light-response types as a percentage of all single units.

(D) Quantification of light-response types as percentage of all light-responsive units. Response types are color coded, as shown in (B).

(E and F) Violin plots of response amplitude in *rd^{1}* + WT retinas (E) for ON-type responses (9.96 ± 0.47 spikes/s; n = 233) and (F) OFF-type responses (6.50 ± 0.44 spikes/s; n = 80) were significantly smaller than in *Gnat1$^{-/-}$* retinas (13.37 ± 0.41 spikes/s, n = 715 and 12.51 ± 0.34 spikes/s, n = 706; p < 0.0001 for both; unpaired t test).

(G and H) Violin plots showing latency to peak response for ON and OFF components of light responses. Latency for ON-type responses in *rd^{1}* + WT cone-transplanted retinas (322.6 ± 17.13 ms) was not significantly different compared with *Gnat1$^{-/-}$* retinas (328.5 ± 10.1 ms; p = 0.77, unpaired t test) but was significantly slower for OFF-type responses (350.1 ± 28.69 ms versus 172.8 ± 7.1 ms; p < 0.0001, unpaired t test).

(I) Peristimulus time histogram of light-responsive units in *Gnat1$^{-/-}$* (black traces; n = 224 units) and *rd^{1}* + WT cone-transplanted retinas (green traces; n = 22 units) in response to a 1-s light step from darkness at six increasing light intensities (time bin = 0.1 s; lights on at time = 0).

(J) Change (Δ) in firing rate over the first 400 ms of the light step plotted as a function of light intensity showed an increase in response amplitude with increasing light intensity, which was not significantly different between the two populations at any intensity investigated (p > 0.99, two-way ANOVA). Mean ± SEM.

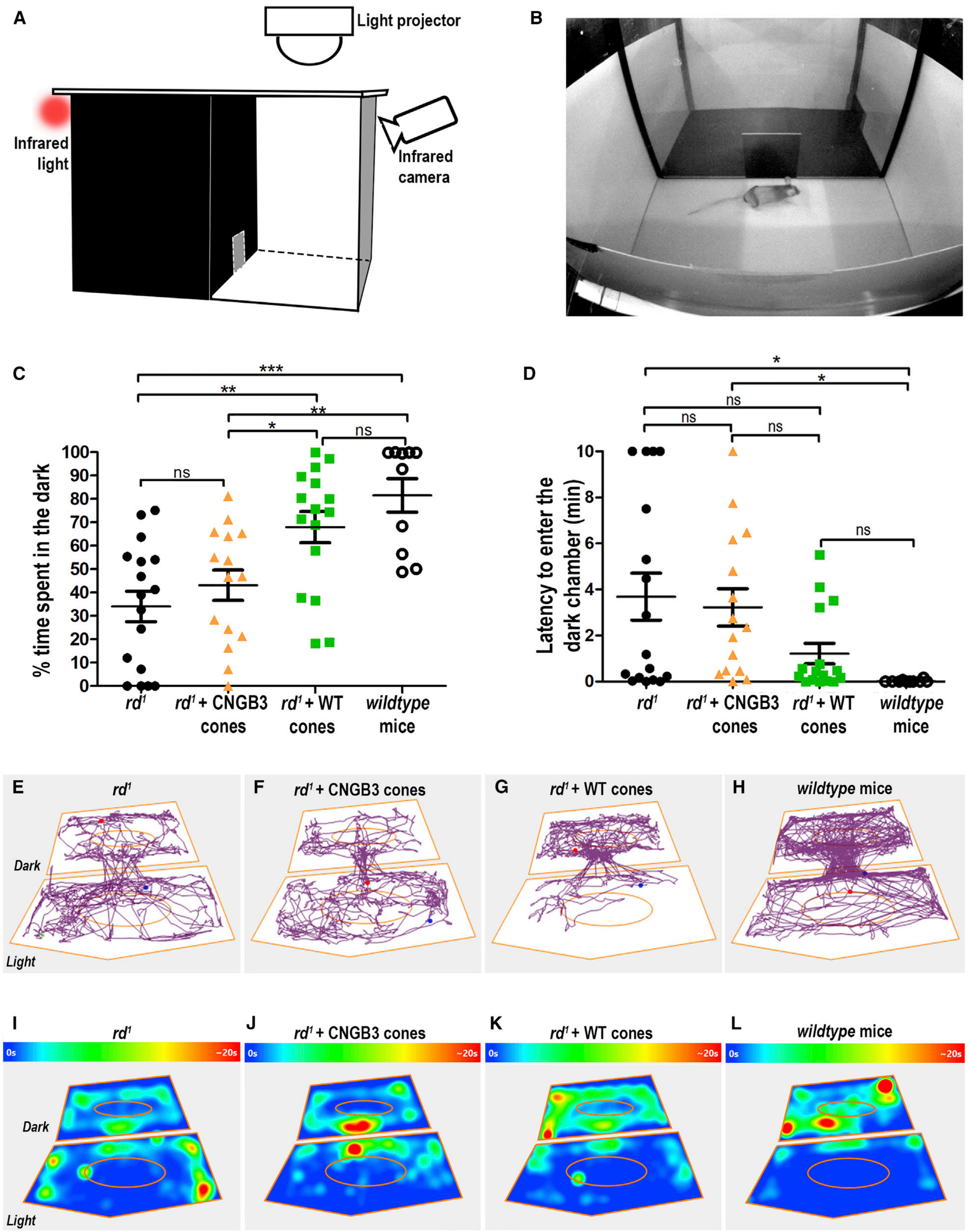

(legend on next page)

CNGB3. Second, there is concern that the transplanted cells may provide neurotrophic factors that have a protective effect on the host photoreceptors and/or inner retinal neurons, delaying degeneration and supporting better function compared with untreated controls (Reh, 2016; West et al., 2020). Here, we show that CNGB3 hiPSC-derived cones survive and mature following transplantation, with no obvious morphological differences when compared with WT hESC-derived cones, but only the latter leads to rescue of retinal and visual function.

Two key features likely underpin the observed rescue. The first is the improved maturation and formation of small, but nonetheless well-organized, OSs by the transplanted cones, a feature not yet seen following the transplantation of dissociated photoreceptors (Barnea-Cramer et al., 2016; Garita-Hernandez et al., 2019; Gonzalez-Cordero et al., 2017; Kruczek et al., 2017). The niche, most likely achieved by the increased number of transplanted cells, appears to be important for OS maturation, similar to OS formation within rosettes of transplanted retinal sheets (Assawachananont et al., 2014; Iraha et al., 2018; Mandai et al., 2017; Shirai et al., 2016). The second is the apparent formation of new synapses between donor human cones and host murine BCs. This is itself remarkable because it was not clear whether human and murine neurons could form heterotypic synaptic connections (Laver and Matsubara, 2017). We show the formation of pre-synaptic ribbons and re-expression of the post-synaptic ON (rod and cone) BC receptor, mGluR6, only in regions of transplantation, together with putative RIBEYE/mGluR6 synaptic complexes. In isolation, these do not prove the formation of a true synapse, and indeed we do not know whether a human/mouse synapse would look similar to a homotypic synapse of either species, but they do strongly indicate an attempt to form a putative synaptic contact. Although others have shown pre-synaptic staining, the only other reports of proximity of pre- and post-synaptic markers are those by Mandai and colleagues (Akiba et al., 2019; Mandai et al., 2017) involving the homotypic transplantation of murine sheets. Our study is the first to show the potential to form functional heterotypic human cone/mouse BC connections.

We observed an encouraging degree of plasticity within the remaining host inner retinal neurons, despite very advanced degeneration. There has been concern that deafferentation-induced remodeling of the inner retina will pose a major barrier to cell replacement strategies for advanced disease (Jones and Marc, 2005; Jones et al., 2003; Reh, 2016; Strettoi, 2015). In contrast, we saw host BCs undergoing widespread reorganization, sending out dendritic projections into the donor cell mass. Neurotransmitters play an important role in many aspects of circuit formation prior to and during synapse formation (Ruediger and Bolz, 2007; Sarin et al., 2018; Tufford et al., 2018). It is plausible that the transplanted cone precursors release glutamate ahead of synapse formation, providing an attractive cue to host BCs. Indeed, pre-synaptic input is required for the formation of the retinal circuit in development, specifically the correct localization of mGluR6 to the dendrites of BCs (Cao et al., 2015), and mGluR6 expression is rapidly downregulated following the loss of pre-synaptic input during photoreceptor degeneration (Puthussery et al., 2009). Consistent with these changes and indicative of the formation of new synapses, we saw widespread re-expression of mGluR6 following transplantation. CNGB3 cones retain the capacity to produce and release glutamate, explaining the same phenotype for both cone populations. Technical reasons prevented the co-staining of host cone BCs, so we used rod BC staining as a corollary but anticipate that cone BCs behave similarly. Encouragingly, central cone BCs have been reported to preferentially survive compared with rod BCs in retinal degeneration (Stefanov et al., 2020). Regardless, cones have been shown to rewire via deafferented rod BCs during degeneration and are able to mediate useful vision (Beier et al., 2017; Strettoi et al., 2004). Further studies are required to dissect and fully characterize the donor cone-host BC connectome that arises following transplantation and how this influences inner retinal processing.

Again, in contrast with anticipated challenges of treating advanced disease, Müller glia extend processes into the cell mass, potentially creating a supportive (Wurm et al., 2011) rather than inhibitory microenvironment. This close interaction is particularly relevant for cone-mediated rescue. A recent transplantation study proposed the use of optogenetically modified cells in order to bypass the requirement for contact between photoreceptor and RPE cells (Garita-Hernandez et al., 2019). However, in contrast with rods, cones rely predominantly on Müller glia to recycle the chromophore all-*trans*-retinal into 11-*cis*-retinal (Wang and Kefalov, 2011). Indeed, unlike previous studies, including those transplanting sheets (Mandai et al., 2017), we did not need to supplement our (RPE-free) retinal preparations with retinaldehyde during MEA recordings, suggesting that chromophore regeneration is sufficient. That not all donor cones make contact with the RPE is, therefore, unlikely to be a significant impediment to successful transplantation. The RPE is still required for phagocytosis of OSs, but this appears to be achieved adequately, at least during the period tested here. Future developments may improve donor cell polarization and interaction with the RPE, and further studies are needed to establish the longer-term survival of transplanted human cones, although we observed no evidence of cell loss over the time frame (at least 4 months) examined here.

Figure 7. Transplantation of purified human WT cones leads to improved visually evoked behavior in rd^1 mice

(A) Schematic of experimental setup showing equally sized light (300 lux) and dark (0 lux at darkest corner) compartments.

(B) Camera view of setup and starting placement of mouse in the arena.

(C) Plot of mean (±SEM) time spent in dark for untreated $rd^1/Foxn1^{nu}$ mice (rd^1) (n = 17 mice), rd^1 + CNGB3 cones (n = 15), rd^1 + WT cones (n = 17), and wild-type mice (n = 10). ***p < 0.001, **p < 0.01, *p < 0.05, one-way ANOVA.

(D) Latency to cross from light to dark for the first time. *p < 0.05, one-way ANOVA.

(E–H) Representative tracking plots from individual animals in each group. Blue and red dots denote mouse's position at start and end of the recording.

(I–L) Heatmap shows mean time spent in any given point across all animals within each experimental group. N.B. points > 20 s (max) are marked as red. Ellipsoids define the central area of each compartment.

We focused particular attention on the electrophysiological responses obtained following transplantation. Indeed, this is the most comprehensive assessment of retinal activity before and after photoreceptor transplantation in the mouse retina to date. We demonstrate a striking robustness of response in those retinas receiving transplants of purified human WT cones: all retinas analyzed showed widespread light-evoked activity that encompassed the extent of the cell mass. This contrasts with previous reports in which the majority of the reported activity derives from a single preparation, with many retinas yielding little or no response. This may be because of the better accessibility to host interneurons afforded by the use of purified cell suspensions. The complexity of the responses recorded is also striking. We could detect nine categories of light-evoked response in *Gnat1*$^{-/-}$ retinas, and eight of these were also present in *rd1/Foxn1*^{nu} mice receiving human WT cones. Such data are not able to determine whether the circuit between transplanted cones and a given RGC is the same as that found in wild-type or indeed *Gnat1*$^{-/-}$ mice. However, it does strongly suggest that human cones are communicating with both ON and OFF BCs, and that a significant degree of inner retinal processing is possible. Notably, response latency fell within the expected limits for synaptic transmission and consistent with the histological data, the responses were blocked by inhibitors of glutamatergic synaptic transmission, showing that human cones make atypical but functional connections with host BCs. Moreover, retinal responses could be evoked with irradiances comparable with normal daylight vision, and these were translated into improvements in visually evoked behavior at light levels that match typical indoor illuminance.

In summary, we provide proof of concept for the restoration of cone-mediated visual function in advanced retinal degeneration following the transplantation of purified cell suspensions of normal hPSC-derived cone photoreceptors and an important validation for the further development of photoreceptor replacement therapy.

STAR★METHODS

Detailed methods are provided in the online version of this paper and include the following:

- KEY RESOURCES TABLE
- RESOURCE AVAILABILITY
  - Lead contact
  - Materials availability
  - Data and code availability
- EXPERIMENTAL MODEL AND SUBJECT DETAILS
  - Animals
  - CNGB3 iPSC line
- METHOD DETAILS
  - Reprograming PBMCs into iPSCs
  - Maintenance of H9 ES and CNGB3 iPS cell lines
  - Differentiation of retinal organoids
  - Production of recombinant AAV viral vector
  - Transplantation
  - Surgery
  - Fundoscopy
  - Immunohistochemistry
  - Image analysis
  - Transmission electron microscopy (TEM)
  - Multi electrode array (MEA)
  - Light stimuli & pharmacology
  - Data analysis
  - Light avoidance
- QUANTIFICATION AND STATISTICAL ANALYSIS

SUPPLEMENTAL INFORMATION

Supplemental information can be found online at https://doi.org/10.1016/j.celrep.2021.109022.

ACKNOWLEDGMENTS

We thank the Institute of Ophthalmology (IoO) vector production and electron microscopy facilities and IoO and Institute of Child Health FACS facilities for technical support. The graphical abstract was generated using BioRender (https://biorender.com/). This work was supported by grants from the Medical Research Council UK (MR/T002735/1 and MR/J004553/1), European Research Council (323147-STEMRD), and Retina UK, and by generous donations from The Miller's Trust, Otto van der Wyck, and F. Gargash.

AUTHOR CONTRIBUTIONS

Conceptualization, J.R., R.A.P., A.G.-C., R.R.A.; methodology, J.R., C.A.P., E.L.W., M.F.M., M.M., D.G., A.J.S., R.A.P., A.G.-C., R.R.A.; investigation, J.R., C.A.P., E.L.W., M.O'H.-W., M.F.M., M.M.K., A.H., M.B., M.F., N.J., M.T., J.W.B.B., A.J.S., R.A.P., A.G.-C.; software, C.A.P., M.M.K.; analysis & interpretation, J.R., C.A.P., M.F.M., M.M.K., M.R., K.P., A.J.S., R.A.P., A.G.-C., R.R.A.; writing – original draft, J.R., C.A.P., A.G.-C., R.A.P.; writing – review & editing, A.J.S., R.A.P., A.G.-C., R.R.A.; funding acquisition, R.A.P., A.G.-C., R.R.A.; supervision, A.J.S., R.A.P., A.G.-C., R.R.A.

DECLARATION OF INTERESTS

The authors declare no competing interests.

Received: July 20, 2020
Revised: January 8, 2021
Accepted: March 31, 2021
Published: April 20, 2021

REFERENCES

Aghaizu, N.D., Kruczek, K., Gonzalez-Cordero, A., Ali, R.R., and Pearson, R.A. (2017). Pluripotent stem cells and their utility in treating photoreceptor degenerations. Prog. Brain Res. *231*, 191–223.

Akiba, R., Matsuyama, T., Tu, H.Y., Hashiguchi, T., Sho, J., Yamamoto, S., Takahashi, M., and Mandai, M. (2019). Quantitative and Qualitative Evaluation of Photoreceptor Synapses in Developing, Degenerating and Regenerating Retinas. Front. Cell. Neurosci. *13*, 16.

Assawachananont, J., Mandai, M., Okamoto, S., Yamada, C., Eiraku, M., Yonemura, S., Sasai, Y., and Takahashi, M. (2014). Transplantation of embryonic and induced pluripotent stem cell-derived 3D retinal sheets into retinal degenerative mice. Stem Cell Reports *2*, 662–674.

Barber, A.C., Hippert, C., Duran, Y., West, E.L., Bainbridge, J.W., Warre-Cornish, K., Luhmann, U.F., Lakowski, J., Sowden, J.C., Ali, R.R., and Pearson, R.A. (2013). Repair of the degenerate retina by photoreceptor transplantation. Proc. Natl. Acad. Sci. USA *110*, 354–359.

Barnea-Cramer, A.O., Wang, W., Lu, S.J., Singh, M.S., Luo, C., Huo, H., McClements, M.E., Barnard, A.R., MacLaren, R.E., and Lanza, R. (2016). Function of human pluripotent stem cell-derived photoreceptor progenitors in blind mice. Sci. Rep. *6*, 29784.

Beier, C., Hovhannisyan, A., Weiser, S., Kung, J., Lee, S., Lee, D.Y., Huie, P., Dalal, R., Palanker, D., and Sher, A. (2017). Deafferented Adult Rod Bipolar Cells Create New Synapses with Photoreceptors to Restore Vision. J. Neurosci. *37*, 4635–4644.

Brown, T.M., Gias, C., Hatori, M., Keding, S.R., Semo, M., Coffey, P.J., Gigg, J., Piggins, H.D., Panda, S., and Lucas, R.J. (2010). Melanopsin contributions to irradiance coding in the thalamo-cortical visual system. PLoS Biol. *8*, e1000558.

Calvert, P.D., Krasnoperova, N.V., Lyubarsky, A.L., Isayama, T., Nicoló, M., Kosaras, B., Wong, G., Gannon, K.S., Margolskee, R.F., Sidman, R.L., et al. (2000). Phototransduction in transgenic mice after targeted deletion of the rod transducin alpha -subunit. Proc. Natl. Acad. Sci. USA *97*, 13913–13918.

Cao, Y., Sarria, I., Fehlhaber, K.E., Kamasawa, N., Orlandi, C., James, K.N., Hazen, J.L., Gardner, M.R., Farzan, M., Lee, A., et al. (2015). Mechanism for Selective Synaptic Wiring of Rod Photoreceptors into the Retinal Circuitry and Its Role in Vision. Neuron *87*, 1248–1260.

D'Orazi, F.D., Suzuki, S.C., and Wong, R.O. (2014). Neuronal remodeling in retinal circuit assembly, disassembly, and reassembly. Trends Neurosci. *37*, 594–603.

Davis, K.E., Eleftheriou, C.G., Allen, A.E., Procyk, C.A., and Lucas, R.J. (2015). Melanopsin-derived visual responses under light adapted conditions in the mouse dLGN. PLoS ONE *10*, e0123424.

Dubis, A.M., Cooper, R.F., Aboshiha, J., Langlo, C.S., Sundaram, V., Liu, B., Collison, F., Fishman, G.A., Moore, A.T., Webster, A.R., et al. (2014). Genotype-dependent variability in residual cone structure in achromatopsia: toward developing metrics for assessing cone health. Invest. Ophthalmol. Vis. Sci. *55*, 7303–7311.

Eleftheriou, C.G., Wright, P., Allen, A.E., Elijah, D., Martial, F.P., and Lucas, R.J. (2020). Melanopsin Driven Light Responses Across a Large Fraction of Retinal Ganglion Cells in a Dystrophic Retina. Front. Neurosci. *14*, 320.

Foik, A.T., Lean, G.A., Scholl, L.R., McLelland, B.T., Mathur, A., Aramant, R.B., Seiler, M.J., and Lyon, D.C. (2018). Detailed Visual Cortical Responses Generated by Retinal Sheet Transplants in Rats with Severe Retinal Degeneration. J. Neurosci. *38*, 10709–10724.

Garita-Hernandez, M., Lampič, M., Chaffiol, A., Guibbal, L., Routet, F., Santos-Ferreira, T., Gasparini, S., Borsch, O., Gagliardi, G., Reichman, S., et al. (2019). Restoration of visual function by transplantation of optogenetically engineered photoreceptors. Nat. Commun. *10*, 4524.

Gonzalez-Cordero, A., West, E.L., Pearson, R.A., Duran, Y., Carvalho, L.S., Chu, C.J., Naeem, A., Blackford, S.J.I., Georgiadis, A., Lakowski, J., et al. (2013). Photoreceptor precursors derived from three-dimensional embryonic stem cell cultures integrate and mature within adult degenerate retina. Nat. Biotechnol. *31*, 741–747.

Gonzalez-Cordero, A., Kruczek, K., Naeem, A., Fernando, M., Kloc, M., Ribeiro, J., Goh, D., Duran, Y., Blackford, S.J.I., Abelleira-Hervas, L., et al. (2017). Recapitulation of Human Retinal Development from Human Pluripotent Stem Cells Generates Transplantable Populations of Cone Photoreceptors. Stem Cell Reports *9*, 820–837.

Gonzalez-Cordero, A., Goh, D., Kruczek, K., Naeem, A., Fernando, M., Kleine Holthaus, S.M., Takaaki, M., Blackford, S.J.I., Kloc, M., Agundez, L., et al. (2018). Assessment of AAV Vector Tropisms for Mouse and Human Pluripotent Stem Cell-Derived RPE and Photoreceptor Cells. Hum. Gene Ther. *29*, 1124–1139.

Hirji, N., Aboshiha, J., Georgiou, M., Bainbridge, J., and Michaelides, M. (2018). Achromatopsia: clinical features, molecular genetics, animal models and therapeutic options. Ophthalmic Genet. *39*, 149–157.

Iraha, S., Tu, H.Y., Yamasaki, S., Kagawa, T., Goto, M., Takahashi, R., Watanabe, T., Sugita, S., Yonemura, S., Sunagawa, G.A., et al. (2018). Establishment of Immunodeficient Retinal Degeneration Model Mice and Functional Maturation of Human ESC-Derived Retinal Sheets after Transplantation. Stem Cell Reports *10*, 1059–1074.

Jones, B.W., and Marc, R.E. (2005). Retinal remodeling during retinal degeneration. Exp. Eye Res. *81*, 123–137.

Jones, B.W., Watt, C.B., Frederick, J.M., Baehr, W., Chen, C.K., Levine, E.M., Milam, A.H., Lavail, M.M., and Marc, R.E. (2003). Retinal remodeling triggered by photoreceptor degenerations. J. Comp. Neurol. *464*, 1–16.

Klimczak, R.R., Koerber, J.T., Dalkara, D., Flannery, J.G., and Schaffer, D.V. (2009). A novel adeno-associated viral variant for efficient and selective intravitreal transduction of rat Müller cells. PLoS ONE *4*, e7467.

Kruczek, K., Gonzalez-Cordero, A., Goh, D., Naeem, A., Jonikas, M., Blackford, S.J.I., Kloc, M., Duran, Y., Georgiadis, A., Sampson, R.D., et al. (2017). Differentiation and Transplantation of Embryonic Stem Cell-Derived Cone Photoreceptors into a Mouse Model of End-Stage Retinal Degeneration. Stem Cell Reports *8*, 1659–1674.

Lamba, D.A., Gust, J., and Reh, T.A. (2009). Transplantation of human embryonic stem cell-derived photoreceptors restores some visual function in Crx-deficient mice. Cell Stem Cell *4*, 73–79.

Laver, C.R.J., and Matsubara, J.A. (2017). Structural divergence of essential triad ribbon synapse proteins among placental mammals – Implications for preclinical trials in photoreceptor transplantation therapy. Exp. Eye Res. *159*, 156–167.

Lin, B., Masland, R.H., and Strettoi, E. (2009). Remodeling of cone photoreceptor cells after rod degeneration in rd mice. Exp. Eye Res. *88*, 589–599.

Lin, B., McLelland, B.T., Aramant, R.B., Thomas, B.B., Nistor, G., Keirstead, H.S., and Seiler, M.J. (2020). Retina Organoid Transplants Develop Photoreceptors and Improve Visual Function in RCS Rats With RPE Dysfunction. Invest. Ophthalmol. Vis. Sci. *61*, 34.

Liu, C., Sherpa, T., and Varnum, M.D. (2013). Disease-associated mutations in CNGB3 promote cytotoxicity in photoreceptor-derived cells. Mol. Vis. *19*, 1268–1281.

MacLaren, R.E., Pearson, R.A., MacNeil, A., Douglas, R.H., Salt, T.E., Akimoto, M., Swaroop, A., Sowden, J.C., and Ali, R.R. (2006). Retinal repair by transplantation of photoreceptor precursors. Nature *444*, 203–207.

Mahato, B., Kaya, K.D., Fan, Y., Sumien, N., Shetty, R.A., Zhang, W., Davis, D., Mock, T., Batabyal, S., Ni, A., et al. (2020). Pharmacologic fibroblast reprogramming into photoreceptors restores vision. Nature *581*, 83–88.

Mandai, M., Fujii, M., Hashiguchi, T., Sunagawa, G.A., Ito, S.I., Sun, J., Kaneko, J., Sho, J., Yamada, C., and Takahashi, M. (2017). iPSC-Derived Retina Transplants Improve Vision in rd1 End-Stage Retinal-Degeneration Mice. Stem Cell Reports *8*, 69–83.

McLelland, B.T., Lin, B., Mathur, A., Aramant, R.B., Thomas, B.B., Nistor, G., Keirstead, H.S., and Seiler, M.J. (2018). Transplanted hESC-Derived Retina Organoid Sheets Differentiate, Integrate, and Improve Visual Function in Retinal Degenerate Rats. Invest. Ophthalmol. Vis. Sci. *59*, 2586–2603.

Meyer, J.S., Shearer, R.L., Capowski, E.E., Wright, L.S., Wallace, K.A., McMillan, E.L., Zhang, S.C., and Gamm, D.M. (2009). Modeling early retinal development with human embryonic and induced pluripotent stem cells. Proc. Natl. Acad. Sci. USA *106*, 16698–16703.

Nakano, T., Ando, S., Takata, N., Kawada, M., Muguruma, K., Sekiguchi, K., Saito, K., Yonemura, S., Eiraku, M., and Sasai, Y. (2012). Self-formation of optic cups and storable stratified neural retina from human ESCs. Cell Stem Cell *10*, 771–785.

Nickerson, P.E.B., Ortin-Martinez, A., and Wallace, V.A. (2018). Material Exchange in Photoreceptor Transplantation: Updating Our Understanding of Donor/Host Communication and the Future of Cell Engraftment Science. Front. Neural Circuits *12*, 17.

Nishiguchi, K.M., Carvalho, L.S., Rizzi, M., Powell, K., Holthaus, S.M., Azam, S.A., Duran, Y., Ribeiro, J., Luhmann, U.F., Bainbridge, J.W., et al. (2015). Gene therapy restores vision in rd1 mice after removal of a confounding mutation in Gpr179. Nat. Commun. *6*, 6006.

Okita, K., Matsumura, Y., Sato, Y., Okada, A., Morizane, A., Okamoto, S., Hong, H., Nakagawa, M., Tanabe, K., Tezuka, K., et al. (2011). A more efficient method to generate integration-free human iPS cells. Nat. Methods *8*, 409–412.

CellPress
OPEN ACCESS

Pearson, R.A., Barber, A.C., Rizzi, M., Hippert, C., Xue, T., West, E.L., Duran, Y., Smith, A.J., Chuang, J.Z., Azam, S.A., et al. (2012). Restoration of vision after transplantation of photoreceptors. Nature *485*, 99–103.

Pearson, R.A., Gonzalez-Cordero, A., West, E.L., Ribeiro, J.R., Aghaizu, N., Goh, D., Sampson, R.D., Georgiadis, A., Waldron, P.V., Duran, Y., et al. (2016). Donor and host photoreceptors engage in material transfer following transplantation of post-mitotic photoreceptor precursors. Nat. Commun. *7*, 13029.

Peng, C., Rich, E.D., and Varnum, M.D. (2003). Achromatopsia-associated mutation in the human cone photoreceptor cyclic nucleotide-gated channel CNGB3 subunit alters the ligand sensitivity and pore properties of heteromeric channels. J. Biol. Chem. *278*, 34533–34540.

Pezzullo, L., Streatfeild, J., Simkiss, P., and Shickle, D. (2018). The economic impact of sight loss and blindness in the UK adult population. BMC Health Serv. Res. *18*, 63.

Procyk, C.A., Eleftheriou, C.G., Storchi, R., Allen, A.E., Milosavljevic, N., Brown, T.M., and Lucas, R.J. (2015). Spatial receptive fields in the retina and dorsal lateral geniculate nucleus of mice lacking rods and cones. J. Neurophysiol. *114*, 1321–1330.

Provencio, I., Rodriguez, I.R., Jiang, G., Hayes, W.P., Moreira, E.F., and Rollag, M.D. (2000). A novel human opsin in the inner retina. J. Neurosci. *20*, 600–605.

Puthussery, T., Gayet-Primo, J., Pandey, S., Duvoisin, R.M., and Taylor, W.R. (2009). Differential loss and preservation of glutamate receptor function in bipolar cells in the rd10 mouse model of retinitis pigmentosa. Eur. J. Neurosci. *29*, 1533–1542.

Reh, T.A. (2016). Photoreceptor Transplantation in Late Stage Retinal Degeneration. Invest. Ophthalmol. Vis. Sci. *57*, ORSFg1-7.

Ruediger, T., and Bolz, J. (2007). Neurotransmitters and the development of neuronal circuits. Adv. Exp. Med. Biol. *621*, 104–115.

Santos-Ferreira, T., Llonch, S., Borsch, O., Postel, K., Haas, J., and Ader, M. (2016). Retinal transplantation of photoreceptors results in donor-host cytoplasmic exchange. Nat. Commun. *7*, 13028.

Sarin, S., Zuniga-Sanchez, E., Kurmangaliyev, Y.Z., Cousins, H., Patel, M., Hernandez, J., Zhang, K.X., Samuel, M.A., Morey, M., Sanes, J.R., and Zipursky, S.L. (2018). Role for Wnt Signaling in Retinal Neuropil Development: Analysis via RNA-Seq and In Vivo Somatic CRISPR Mutagenesis. Neuron *98*, 109–126.e8.

Schroeder, M.M., Harrison, K.R., Jaeckel, E.R., Berger, H.N., Zhao, X., Flannery, M.P., St Pierre, E.C., Pateqi, N., Jachimska, A., Chervenak, A.P., and Wong, K.Y. (2018). The Roles of Rods, Cones, and Melanopsin in Photoresponses of M4 Intrinsically Photosensitive Retinal Ganglion Cells (ipRGCs) and Optokinetic Visual Behavior. Front. Cell. Neurosci. *12*, 203.

Seiler, M.J., and Aramant, R.B. (2012). Cell replacement and visual restoration by retinal sheet transplants. Prog. Retin. Eye Res. *31*, 661–687.

Shirai, H., Mandai, M., Matsushita, K., Kuwahara, A., Yonemura, S., Nakano, T., Assawachananont, J., Kimura, T., Saito, K., Terasaki, H., et al. (2016). Transplantation of human embryonic stem cell-derived retinal tissue in two primate models of retinal degeneration. Proc. Natl. Acad. Sci. USA *113*, E81–E90.

Singh, M.S., Charbel Issa, P., Butler, R., Martin, C., Lipinski, D.M., Sekaran, S., Barnard, A.R., and MacLaren, R.E. (2013). Reversal of end-stage retinal degeneration and restoration of visual function by photoreceptor transplantation. Proc. Natl. Acad. Sci. USA *110*, 1101–1106.

Singh, M.S., Balmer, J., Barnard, A.R., Aslam, S.A., Moralli, D., Green, C.M., Barnea-Cramer, A., Duncan, I., and MacLaren, R.E. (2016). Transplanted photoreceptor precursors transfer proteins to host photoreceptors by a mechanism of cytoplasmic fusion. Nat. Commun. *7*, 13537.

Stefanov, A., Novelli, E., and Strettoi, E. (2020). Inner retinal preservation in the photoinducible I307N rhodopsin mutant mouse, a model of autosomal dominant retinitis pigmentosa. J. Comp. Neurol. *528*, 1502–1522.

Strettoi, E. (2015). A Survey of Retinal Remodeling. Front. Cell. Neurosci. *9*, 494.

Strettoi, E., Mears, A.J., and Swaroop, A. (2004). Recruitment of the rod pathway by cones in the absence of rods. J. Neurosci. *24*, 7576–7582.

Tufford, A.R., Onyak, J.R., Sondereker, K.B., Lucas, J.A., Earley, A.M., Mattar, P., Hattar, S., Schmidt, T.M., Renna, J.M., and Cayouette, M. (2018). Melanopsin Retinal Ganglion Cells Regulate Cone Photoreceptor Lamination in the Mouse Retina. Cell Rep. *23*, 2416–2428.

Wang, J.S., and Kefalov, V.J. (2011). The cone-specific visual cycle. Prog. Retin. Eye Res. *30*, 115–128.

Wang, Y., Macke, J.P., Merbs, S.L., Zack, D.J., Klaunberg, B., Bennett, J., Gearhart, J., and Nathans, J. (1992). A locus control region adjacent to the human red and green visual pigment genes. Neuron *9*, 429–440.

West, E.L., Ribeiro, J., and Ali, R.R. (2020). Development of Stem Cell Therapies for Retinal Degeneration. Cold Spring Harb. Perspect. Biol. *12*, a035683.

Wong, K.Y. (2012). A retinal ganglion cell that can signal irradiance continuously for 10 hours. J. Neurosci. *32*, 11478–11485.

Wong, K.Y., Dunn, F.A., Graham, D.M., and Berson, D.M. (2007). Synaptic influences on rat ganglion-cell photoreceptors. J. Physiol. *582*, 279–296.

Wurm, A., Pannicke, T., Iandiev, I., Francke, M., Hollborn, M., Wiedemann, P., Reichenbach, A., Osborne, N.N., and Bringmann, A. (2011). Purinergic signaling involved in Müller cell function in the mammalian retina. Prog. Retin. Eye Res. *30*, 324–342.

Zhang, D.-Q., Zhou, T.-R., and McMahon, D.G. (2009). Structural and Functional Preservation of Dopaminergic Amacrine Cells in Retinal Degeneration. Invest. Ophthalmol. Vis. Sci. *50*, 5684.

Zhao, X., Stafford, B.K., Godin, A.L., King, W.M., and Wong, K.Y. (2014). Photoresponse diversity among the five types of intrinsically photosensitive retinal ganglion cells. J. Physiol. *592*, 1619–1636.

Zhong, X., Gutierrez, C., Xue, T., Hampton, C., Vergara, M.N., Cao, L.H., Peters, A., Park, T.S., Zambidis, E.T., Meyer, J.S., et al. (2014). Generation of three-dimensional retinal tissue with functional photoreceptors from human iPSCs. Nat. Commun. *5*, 4047.

Zhu, J., Cifuentes, H., Reynolds, J., and Lamba, D.A. (2017). Immunosuppression via Loss of IL2rγ Enhances Long-Term Functional Integration of hESC-Derived Photoreceptors in the Mouse Retina. Cell Stem Cell *20*, 374–384.e5.

STAR★METHODS

KEY RESOURCES TABLE

REAGENT or RESOURCE	SOURCE	IDENTIFIER
Antibodies		
Anti-GFP antibody (FITC)	Abcam	ab6662, RRID:AB_305635
PKC-α	Santa Cruz	sc10800, RRID:AB_2168560
Mouse Cone Arrestin	Milipore	ab15282, RRID:AB_1163387
Human Cone Arrestin (ARRESTIN3)	Novus	NBP1-19629, RRID:AB_2243059
Rhodopsin	Sigma Aldrich	O4886, RRID:AB_260838
M-opsin	Milipore	AB5405, RRID:AB_177456
HuC/HuD	ThermoFisher	A-21271, RRID:AB_221448
mGluR6	Sigma Aldrich	SAB4501324, RRID:AB_10761963
VGLUT	Sigma Aldrich	AB5905, RRID:AB_2301751
CtBP2 (Ribeye)	BD Biosciences	612044, RRID:AB_399431
Calretinin	Abcam	ab702, RRID:AB_305702
Human Nuclei	Millipore	MAB1281, RRID:AB_94090
Peripherin2	Gift from Gabriel Travis	Not Applicable
Synaptophysin	Sigma Aldrich	S5768, RRID:AB_477523
GFAP	DAKO	Z0334, RRID:AB_10013382
c-Myc	Cell Signaling Technology	5605, RRID:AB_1903938
LIN28A	Cell Signaling Technology	3695, RRID:AB_2135033
Nanog	Cell Signaling Technology	4903, RRID:AB_10559205
Oct-4A	Cell Signaling Technology	2840, RRID:AB_2167691
Sox2	Cell Signaling Technology	3579, RRID:AB_2195767
Bacterial and virus strains		
AAV.ShH10.L/Mopsin.eGFP	Viral Production Facility, UCL Institute of Ophthalmology	Gonzalez-Cordero et al., 2017
Biological samples		
Patient blood	Moorfields Eye Hospital	N/A
Chemicals, peptides, and recombinant proteins		
Ames	Sigma Aldrich	A1420
Sodium Bicarbonate	Sigma Aldrich	S6014
L-AP4: L-(+)-2-Amino-4-phosphonobutyric acid	AbCam	ab120002
DNQX: 6,7-Dinitroquinoxaline-2,3(1H,4H)-dione	Sigma Aldrich	D0540
D-AP5: D(−)-2-Amino-5-phosphonopentanoic acid	Sigma Aldrich	A8054
Critical commercial assays		
Neurosphere Dissociation Kit (P)	Miltenyi Biotec	130-095-943
P3 Primary Cell 4D-NucleofectorTM X Kit L	Lonza	V4XP-3024
Experimental models: Cell lines		
Human: H9 ES cells	WiCell	WA09
Human: CNGB3 iPS cells	This paper	N/A
Experimental models: Organisms/strains		
Mouse: Crl:NU(NCr)-Foxn1^{nu}	Charles River	490
Mouse: C3H/HeJ (rd1)	The Jackson Laboratory	000659

(*Continued on next page*)

Continued

REAGENT or RESOURCE	SOURCE	IDENTIFIER
Mouse: *rd1/Foxn1*^{nu}	This paper	N/A
Mouse: C57BL/6J	The Jackson Laboratory	000664
Mouse: *Gnat1*$^{-/-}$	Janis Lem, Tufts Medical Centre	Calvert et al., 2000
Recombinant DNA		
Plasmid: L-MYC and LIN28 episonal insert (pCXLE-hUL)	Okita et al., 2011	Addgene plasmid #27080
Plasmids: SOX2 and KLF4 episomal insert (pCXLE-hSK)	Okita et al., 2011	Addgene plasmid #27078
Plasmids: OCT3/4 episomal insert (pCXLE-hOCT3/4-shp53-F)	Okita et al., 2011	Addgene plasmid #27077
Software and algorithms		
Prism (versions 5 and 7)	GraphPad	GraphPad
ANY-maze	Stoelting Europe	N/A
Adobe Photoshop CS6	Adobe Systems	CS6
ImageJ	NIH	https://imagej.nih.gov/ij/
Multi-Channel Data Suite	Multi-Channel Systems	https://www.multichannelsystems.com/
Offline Sorter V4.4.2	Plexon Inc.	https://plexon.com/
NeuroExplorer V5	Nex Technologies	http://www.neuroexplorer.com/
MATLAB R2019B	The Mathworks	https://www.mathworks.com/
CorelDraw 2020 V22.1.0.517	The Corel Corporation	https://www.coreldraw.com/en/

RESOURCE AVAILABILITY

Lead contact

Further information and requests for animal strains or cell lines should be directed to and will be fulfilled by the Lead Contact, Robin Ali (robin.ali@kcl.ac.uk), upon execution of a suitable Materials Transfer Agreement.

Materials availability

All unique/stable reagents generated in this study will be made available on request but may require a payment and/or a completed Materials Transfer Agreement if there is potential for commercial application. *Rd1/Foxn1*^{nu} mice will be provided directly; in the event of high demand, and presuming the strain will be accepted, the Lead Contact will deposit the strain in the Jackson Laboratories collection

Data and code availability

The datasets supporting the current study have not been deposited in a public repository because the authors are undertaking further analysis and any outputs arising from these will be published in due course but are available from the corresponding author on reasonable request.

EXPERIMENTAL MODEL AND SUBJECT DETAILS

Animals

Details of mouse lines, sources and relevant citations can be found in the Key resources table. Animals were maintained on a standard 12 hour light-dark cycle. Mice received food and water ad lib and were provided with fresh bedding and nesting weekly. Crl:NU(NCr)-Foxn1^{nu} and rd1/Crl:NU(NCr)-Foxn1^{nu} mice are immunocompromised and were maintained under the same conditions but with autoclaved cages, bedding and water, and irradiated food was provided.

To generate *rd1/Foxn1*^{nu} animals, we crossed *C3H/HeN* mice with Crl:NU(NCr)-*Foxn1*^{nu} mice. Animals were genotyped for the *Foxn1*^{nu} gene based on phenotype (lack of hair) and PCR analysis of DNA (ear clip) was used for *PDE6*β gene (*rd1*). Double homozygote males were bred with *PDE6*$\beta^{-/-}$ females that were heterozygote for *Foxn1*^{nu} (Homozygous nude females are not effective breeders).

Animals were used at 3 months of age (11-14 weeks) and kept for a further 3 to 4 months post-transplantation, under the conditions described above. Some animals received treatment in one eye, while others received the same treatment in both eyes. Both males and females were used as recipients without discrimination. No immunosuppression was administered. Animals were sacrificed by cervical dislocation.

All experiments were conducted in accordance with the United Kingdom Animals (Scientific Procedure) Act of 1986 and Policies on the Use of Animals and Humans in Neuroscience Research.

CNGB3 iPSC line

Following informed consent, peripheral blood was taken from a male individual, aged 40 years at the time of donation, with achromatopsia due to a homozygous deletion (c.1148delC) in the *CNGB3* gene. Peripheral blood mononuclear cells (PBMCs) were reprogramed into iPSCs using established methodology: (https://cambridgebrc.nihr.ac.uk/expandables/hipsc-core-facility/human-induced-pluripotent-stem-cells-protocols-v10/). Karyotype analysis was performed and analyzed by TDL Genetics. The study followed the tenets of the Declaration of Helsinki and was approved by the Moorfields and Whittington Hospitals' local Research Ethics Committees and the NRES Committee London Riverside Ethics Committee (REC 11/H0721/13).

METHOD DETAILS

Reprograming PBMCs into iPSCs

On day 0, 2x 10^6 PBMCs were defrosted and added to 10ml of prewarmed (37°C) Stemspan 3000 with Penicillin/ Streptomycin (SS Medium). Following centrifugation at 300 g for five minutes, the pellet of cells was resuspended in Expansion Medium (EM) and plated in one well of a 12-well plate. On day 3, cells were collected, centrifuged, resuspended in 2ml of EM Medium (see medium preparation) and plated in one well of a 12-well plate. Cells were left to expand in expansion medium (EM, see Media Preparation for details) for a further 3 days prior to nucleofection (6 days in total) prior to reprograming.

On day 6, medium containing the cells was collected into a 15ml falcon tube. 200,000 cells were transferred into a new tube with 12ml of PBS and centrifuged at 300 g for 5 minutes. Lonza P3 Nucleofection supplement was added to P3 buffer (P3 buffer), from P3 Primary Cell 4D-Nucleofector X Kit L (Lonza, V4XP-3024), according to manufacturer instruction (20μl/electroporation reaction). Addgene episomal plasmids pCXLE-hUL [pCXLE-hUL was a gift from Shinya Yamanaka (Addgene plasmid # 27080; http://www.addgene.org/27080; RRID:Addgene_27080)], pCXLE-hSK [pCXLE-hSK was a gift from Shinya Yamanaka (Addgene plasmid # 27078; http://www.addgene.org/27078/; RRID:Addgene_27078)] and pCXLE-hOCT3/4-shp53-F [pCXLE-hOCT3/4-shp53-F was a gift from Shinya Yamanaka (Addgene plasmid # 27077; http://www.addgene.org/27077/; RRID:Addgene_27077)] (Okita et al., 2011) were added to P3 buffer (0.33 μg each). Following centrifugation, the cell pellet was resuspended in the P3 buffer containing Yamanaka's plasmids. Cells were then transferred to the cuvette strip (Lonza, V4XP-3024) and electroporated using program EO-115 of Amaxa 4D-Nucleofector System (Lonza). After electroporation cells were kept at room temperature for 5 minutes. 80μL of Roswell Park Memorial Institute (RPMI) 1640 (GIBCO, #11875085) was added to the cuvette strip and it was placed at 37°C for 10 minutes. Cells were then added to 1 well of a 6-well plate (Corning®Costar, 3516), previously coated with Geltrex (1:100), in 2ml of EM. Cells were kept in EM until day 8. From day 8 to day 10 cells were fed daily with a 1:1 mix of Essential 8 Medium (E8 Medium; Invitrogen, #A1517001) and EM. After day 10 cells were fed daily with E8 Medium.

Maintenance of H9 ES and CNGB3 iPS cell lines

All cells were kept at 37°C with 5% CO_2. The human embryonic stem cell (hESC) line, H9 (WA09, Wicell) and CNGB3 iPSC lines were maintained as previously described (Gonzalez-Cordero et al., 2017). Cell were maintained in E8 Medium, in 6-well tissue culture plates (Corning®Costar, 3516) coated with Geltrex (Invitrogen, A1413302). The media was changed (2 mL per well) every day until cells presented 90%–100% confluency. One well per plate was used to split, while the remaining 5 went forward for differentiation. Cells were split twice a week, when 80%–90% confluent. After PBS wash 1ml of pre-warmed Versine (GIBCO, 15040033) was added for 5 minutes at 37°C. Versine was carefully aspirated and 2ml of E8 was added before lifting the cells and transferring to a falcon tube. 200,000 were plated in each well of 6-well plate containing 2ml of E8 and 2μL Rock Inhibitor (Live Technologies, 1254).

Differentiation of retinal organoids

Cells were differentiated as we have previously reported (Gonzalez-Cordero et al., 2017). Briefly, when 90%–100% confluent, Essential 6 Medium (Invitrogen, A1516401) was added for 2 days. This change marks differentiation day 1 (dd1-2). On dd3, cells were transferred to Proneural Induction Medium (PIM). Hereafter, media was changed every 2 days. Cultures remain in PIM until retinal organoids start to develop, for a maximum of 40 days. When retinal organoids appeared, these were individually dissected and transferred to low binding 96 well plates (Nuclon Sphera) for 3D culture in Retinal Differentiation Medium (RDM), until dd42. At dd42, factors were added to the Medium (RDM + Factors). After 70 days in culture individual retinal organoids were transferred to low binding 24 well tissue culture plates (Corning®Costar, 734-1584) and Retinoic Acid (RA) was added fresh to the Medium at a concentration of 1μg/ml at each feeding. By dd90, RDM+Factors+RA Medium was replaced by a different formulation of Retinal Differentiation Medium (RDM90). RA was added fresh to RDM90 before use at a concentration of 0.5μg/ml.

Production of recombinant AAV viral vector

All adeno-associated viral vectors used were of the serotype ShH10 (Klimczak et al., 2009) carrying an addition tyrosine to phenylalanine mutation at position 445 of the VP proteins. The transgene encoded was a GFP reporter under the control of a previously described 2.1PR promoter (Wang et al., 1992), which specifically labels L/M opsin cone photoreceptors, a fidelity we have

reproduced in human ESC-derived cone photoreceptors (Gonzalez-Cordero et al., 2017, 2018). A pD10/2.1PRL/Mopsin promoter-GFP plasmid containing AAV-2 inverted terminal repeat (ITR) was used to generate ShH10(Y445F) L/Mopsin.GFP vectors. Recombinant AAV2/2 serotype particles were produced through a previously described triple transient transfection method HEK293T cells (Nishiguchi et al., 2015). AAV-ShH10(Y445F) serotype was bound to an AVB Sepharose column (GE Healthcare) and eluted with 50 mM Glycine pH2.7 into 1 M Tris pH 8.8. Vectors were washed in 1 × PBS and concentrated to a volume of 100–150 μl using Vivaspin 4 (10 kDa) concentrators. Viral genome titers were determined by quantitative real-time PCR using a probe-based assay binding the SV40 poly-adenylation signal. Amplicon-based standard series of known amounts were used for sample interpolation. Final titers are expressed as vg/mL.

SV40 Forward primer: 5′-Agcaatagcatcacaaatttcacaa-3′.
SV40 Reverse primer: 5′-AGATACATTGATGAGTTTGGACAAAC-3′.
SV40 Probe: FAM-5′-AGCATTTTTTTCACTGCATTCTAGTTGTGGTTTGTC-3′

Neuroretinal vesicles were infected with approximately $1.2x10^{11}$ viral particles per well in RDM.

Transplantation

Cell preparation

Retinal organoids (minimum 120 organoids) were dissociated at week 17 (±2 wks) of differentiation, using the papain-based Neurosphere Dissociation Kit (Miltenyi Biotec, 130-095-943) according to manufacturer's instructions. Prewarmed enzyme solution was added to retinal organoids (1mL/30 organoids) for 15 minutes at 37°C. Mechanical trituration was performed using a 1000 μL pipette tip and cells were centrifuged at 900 rpm for 7 minutes. Cells were resuspended at 20×10^6 cells/ml in FACS buffer (66% MEM-E HEPES, 33% HBSS (+Mg^{2+}, -Ca^{2+}), 1% FBS, Dnase I (10 μL/ml)) and passed through a 35-μm mesh cell strainer (BD Falcon, UK; cat no 352235). GFP+ cells were sorted by fluorescence activated cell sorting (FACS) on cell sorter Moflo XDP (Beckman Coulter, California). Coherent Sapphire blue laser (150mW 488nm) was used to excite GFP. GFP signal was collected in FL-1 channel through a 530/40 band pass filter and FL-3 channel fitted with a 613/20 band pass filter was used to separate GFP+ cells from auto-fluorescence background. Gating was set to select for purity of GFP. A light scatter gate was drawn in the FSC versus SSC plot to exclude debris and clumps and include viable cells. Cells in this gate were displayed as SSC versus SSC-W to select only single cells. Single and viable cells were then analyzed in a FL3-Log versus FL1-Log plot and a final gate was drawn on viable GFP+ cells for cell sorting and sorted with a 100 nm nozzle at 20 PSI sheath pressure. GFP+ cone photoreceptor cells were collected in 20% FBS in DMEM and centrifuged (7 minutes, 900rmp) before being resuspended to a final concentration of 0.5×10^6 in1.5 μl of EBSS/0.05%DNaseI solution. Sorted cell suspensions were at least 90%, and typically 98% - 99.9%, GFP+ according to FACS-based purity checks. Although GFP levels detectable by imaging varied between individual cells (due to virus expression levels), immunocytochemistry for human CONE ARRESTIN confirmed that the sorted cells were a pure population of cones (> 98% of viable cones were immuno-positive for hCARR; viability of the sorted population was > 90% at time of transplantation; Figure 1).

Surgery

All surfaces were wiped with 70% ethanol prior to surgery. 3 month old *rd1/Foxn1^{nu}* mice were anesthetized by intraperitoneal injection (10mL/Kg) of a mixture of a mixture of Dormitor (1 mg/ml medetomidine hydrochloride; Pfizer Pharmaceuticals), ketamine (100 mg/ml; Fort Dodge Animal Health), and sterile water in the ratio 5:3:42. Tropicamide (1%; Bausch & Lomb) was used to dilate the pupils and topical anesthetic was applied (Tetracaine). Eyes were kept moist by using Viscotears (Novartis Pharmaceuticals UK Ltd). Surgery was performed under direct visual control using an operating microscope (Zeiss). A sterile 34-gauge hypodermic needle was used to make a small puncture to the anterior chamber to relieve pressure in the orbit. The same needle was used to make a new transcleral entry in the posterior orbit and slowly inject 1.5μl of cell suspension into the sub-retinal space. The same region of the eye was targeted for all injections. Significant care was taken not to rupture the very thin remaining neural retina and the needle was left in place for ∼20 s to allow for re-equilibration of intraocular pressure before slowly withdrawing. Anaesthesia was then reversed using same amount of Antisedan, 10mL/Kg, (atipamezole hydrochloride 0.10 mg/ml, Pfizer Pharmaceuticals, Kent UK), with the mice placed on heat mats until fully recovered.

Fundoscopy

Mice were anaesthetised to minimize motion blurring. Retinal GFP fluorescence was imaged using a retinal imaging microscope (Micron 3; Phoenix technology group) fitted with a lens optimized to the murine eye, a filter set to detect GFP (Excitation: 469 ± 18nm, Emission: 500nm Long Pass) and associated software. All bright field images were taken at Gain = 0, no long exposure. The default settings for fluorescent images was Gain = 10, Long exposure = 10 but these were varied subjectively to best visualize the extent of the GFP+ area. A coupling medium (Viscotears, Novartis Pharmaceuticals) was applied between the lens and the corneal surface to minimize refraction. After imaging mice were either recovered as previously described. Fundoscopy was used to assess the presence of a substantial GFP+ cell mass. A transplant was considered successful if the GFP+ cell mass occupied an area at least 10% of the retina visible through fundoscopy (representative example shown in Figures S3G and S3H). Rarely, we saw evidence of transplantation failure, where no or only very sparse, single GFP+ cells were seen. These animals were not

processed for MEA or behavioral analysis. Moreover, a small number of animals were retrospectively removed from the dataset upon histological examination, which revealed the injections having been inadvertently introduced into the intravitreal cavity (this cannot be determined by fundoscopy in the living animal). > 70% of transplantations were classified as successful.

Immunohistochemistry

Mice were euthanized by cervical dislocation. Eyes were dissected to remove the cornea, iris, and lens. All samples were fixed for 1 hour in 4% paraformaldehyde (PFA), cryopreserved in 20% sucrose (30 minutes at room temperature or overnight at 4°C) and embedded in OCT (TissueTek) and frozen. Samples were then cryosectioned at 12μm thickness and stored at −20°C. Cryosections were blocked for 1 hour at room temperature with 3% goat serum and 1% bovine serum albumin (BSA) in PBS with 0.1% Triton X-100. For anti-mGluR6 antibody heat induced antigen retrieval was performed with in target retrieval solution, citrate pH 6.1 (S1700, DAKO), 3 minutes in 600watts microwave. Primary antibodies were incubated at 4°C overnight. Cryosections were incubated with secondary antibody Alexa Fluor 488, 546 or 633 (Invitrogen-Molecular Probes) at a 1:500 dilution, for 2 hours at room temperature, and counterstained with DAPI (1:1000, Sigma-Aldrich).

Images were acquired by confocal microscopy (Leica DM5500Q). A series of XY optical sections were taken at approximately 1.0μm steps, throughout the depth of the section, typically comprising 19 to 21 sections/stack. Stacks were then used to generate maximum intensity projection (MIP) images using LAS AF image software. All confocal fluorescent images shown are MIP images, unless otherwise stated (whereupon single section images may be shown to improve clarity and/or confirm co-localization). In order to achieve maximum resolution and clarity over a large area, in some figure panels the images shown are composite images of two or more image stacks "stitched" together using image software, and then cropped to a suitable size. This may result in very minor vertical or horizontal misalignments in some places. Panels involving composite images are Figures 1G and 1H, 2D and its associated ROI, 3A, and S4A, S4B, and S4D.

Image analysis

To quantify nuclear size, HNA expression, Mopsin expression and frequency of PRPH2+ segment-like generation eyes bearing comparable-sized cell masses from either cell line (WT and CNGB3) were used. For very large cell masses (one for each group), 2 areas were identified. Therefore, n = 6 areas, N = 4 animals, for WT cones; n = 5 areas, N = 4 animals, for CNGB3 cones).

Outer segment quantification was performed by manual selection of segment-like structures from confocal image files. Maximum intensity projection stacks were taken at the site of transplantation (20 to 22 stacks at 1mm step intervals) as identified by GFP+ cell mass. Post-image acquisition, channels were separated (GFP, green, DAPI, blue; PRPH2, red). Region of cell mass was demarcated, and images were masked by a lab member not involved in the project. Number of nuclei and number of PRPH2+ segment-like buds were quantified manually and % of PRPH2+/nuclei in cell mass was calculated for each image. Images were then unmasked.

Quantification of the proportion of cells expressing Mopsin/HNA and HNA/Dapi, was performed by manual assessment of the confocal image files in a fully blinded manner. Maximum intensity projection mini-stacks (3 stacks at 1mm step intervals, taken in the middle of the z stack depth) were taken at the site of transplantation, as identified by presence of GFP+ cell mass. Post-image acquisition, channels were separated (GFP, green; DAPI, blue; Mopsin or HNA, red). The region of the GFP+ cell mass was demarcated, and images were masked by a lab member not involved in the project. The proportion of nuclei that colocalized with Mopsin or HNA, as appropriate, were quantified manually. Images were then unmasked.

mGluR6 signal intensity was determined by measuring the integrated density values for a standardized ROI taken at the site of transplantation to encompass outer edge of INL and cell mass and an equivalent ROI taken at the opposite side of the same eye away from the site of transplantation. Images were acquired in the same imaging session using identical microscope settings. 2-3 ROIS were taken for each eye and an average value determined. Expression changes are shown as a fold-change in average integrated signal intensity per retina.

Nuclear size was determined by selecting cells at random and measuring along the longest axis using ImageJ.

Transmission electron microscopy (TEM)

Transplanted eyes with large cell mass (as determined by fundoscopy) were selected and the nasal area was stitched to allow orientation of the sample. Eyes were dissected to remove the cornea, iris, and lens. Samples were fixed for 24 hours in Karnovky fixative (1% PFA, 3% glutaraldehyde, 0.08M sodium sodium cacodylate-HCL). Samples were osmicated in 1% aqueous solution of osmium tetroxide, for 2 hours in the dark and then dehydrated through ascending ethanol series (30% - 100%, 10 minutes each solution). To conclude dehydration, samples were incubated in propylene oxide (total of 30 minutes with 3 changes of solution) and left overnight with rotation in a 1:1 mixture of propylene oxide and araldite resin. The samples were then changed into 100% araldite resin and left for 6 hours with rotation. Samples were embedded in new araldite resin and cooked for 24 hours at 60°C. Semi-thin (700 nm) and ultrathin (50-70 nm) sections were cut using a Leica ultracut S microtome with a diamond knife (Diatome histoknife Jumbo). Semi-thin sections were collected onto slides, stained with 1% alcoholic toluidine blue and evaluated under light microscope. The region of transplantation was identified by retinal detachment and displaced RPE. Ultrathin sections were collected on to 100 mesh copper grids (Agar Scientific), counter-stained with lead citrate and imaged using a JEOL 1400 Transmission Electron Microscope. When imaging ultrathin sections, the area of injection was identified by the presence of a retinal detachment, displaced RPE and by the presence of several cytoplasm pockets containing mitochondria (Inner Segments).

Multi electrode array (MEA)

In-vitro electrophysiology

Five *Gnat1*$^{-/-}$, six *rd1/FoxN1*^{nu} + WT mice, five *rd1/FoxN1*^{nu} + CNGB3 and five untreated *rd1/FoxN1*^{nu} mice were euthanized by cervical dislocation 3 months post-transplantation. Eyes were immediately enucleated, and retinal isolation was performed in the dark in warm carboxygenated (95% O_2-5% CO_2) AMES media supplemented with 1.9 g/L sodium bicarbonate (Sigma Aldrich, UK). The retina was incised four times in a Maltese cross motif and mounted onto a perforated Multi Electrode Array (120pMEA100/30iR-ITO or USB-MEA60; MultiChannel Systems, Reutlingen, Germany) with the ganglion cell layer facing down onto the electrodes. Three *rd1* + WT and x1 *rd1* + CNGB3 retinas were recorded on 60-channel MEA. All other recordings were performed on 120-channel (x3 *rd1* + WT; x4 *rd1* + CNGB3; x5 *Gnat1*$^{-/-}$; x5 *rd1* untreated). All Figures show data from the 120-channel system. For injected animals, GFP expressing regions of the retinal cell mass were placed centrally over the electrodes in order to maximize the recording area from transplanted cones. A platinum harp (0.5g) bearing a framework of parallel silicon strings was used to hold the retina in place and keep it stable during recording. The MEA chamber was then mounted into the amplifier (MEA2100-120 head stage; Multi Channel Systems). Electrophysiological signals were digitised and recorded with a sampling frequency of 20 kHz using Multi Channel Experimenter (Multi Channel Systems). Prior to recording, the retina was allowed to rest for 30 minutes of dark adaptation and to allow for neuronal activity to stabilize. To preserve physiological conditions, the tissue was perfused with carboxygenated AMES (PPS2; Multi Channel Systems) and maintained at 36°C (TC02 controller; Multi Channel Systems) throughout the duration of the experiment. No Retinaldehyde was supplemented during these experiments.

Presentation of visual stimuli

Light stimuli were designed using MC_Stimulus II (Multi Channel systems), which programmed a T-Cube LED Driver (Thorlabs; Germany) to control a mounted Cyan LED (λ_{max} = 505nm; M505L4; Thorlabs). A light guide was used to project light from the LED onto the retina from above (Irradiance at retinal surface = 4.21×10^{15} photons cm^{-2} s^{-1}). All light measurements were recorded using a calibrated spectroradiometer (SpectroCal; Cambridge Research Instruments, UK).

Light stimuli & pharmacology

1 s flashes

Full-field 1 s light steps were presented from darkness with a 20 s interstimulus interval and repeated 20 times.

Sensitivity

In two *Gnat1*$^{-/-}$ and two *rd1/FoxN1*^{nu} + WT retinas, 20 repeats of a full-field 1 s light steps with a 20 s interstimulus interval, were presented at six increasing light intensities from a dark-adapted background (irradiance = 4.21×10^{15} photons$\cdot$cm$^{-2}\cdot$s^{-1}). The stimulus sequence started from the lowest light level and ended at the highest.

Pharmacology

The glutamatergic blockers L (+)-2-amino-4-phosphonobutyrate (L-AP4) (group III metabotropic glutamate receptor agonist) (50 μM), 6, 7-dinitroquinoxaline-2, 3-dione (DNQX) (AMPA/kainate receptor antagonist) (40 μM), and d-2-amino-5-phosphonovalerate (d-AP5) (NMDA receptor antagonist; all from Sigma Aldrich, UK) (40 μM) were added to the AMES media, to block synaptic input from outer retinal photoreceptors and identify the origin of light responses in retinal explants. Under these conditions, we repeated the 1 s light pulses described above. Following this protocol, the drugs were washed out and the MEA chamber was cleared with AMES media (Sigma Aldrich, UK) for 1 hour before repeating the same 1 s light stimulation protocol.

Data analysis

Spike sorting

Offline, neural waveforms were processed using Offline Sorter (v4.4.2; Plexon). Cross-channel artifacts were identified and removed, and then each channel was analyzed separately. For each channel, single-unit spikes were detected and categorized on the basis of the spike waveform via a principal component analysis, whereby distinct clusters of spikes were readily identifiable and showed a clear refractory period in their interspike interval distribution (> 1ms) (Figure S5). Single-unit data were subsequently sent too and stored in NeuroExplorer (v5.112; Nex Technologies, MA) in preparation for further analysis.

Identification of light responses

Spike sorted data in Neuroexplorer files was analyzed by custom written MATLAB codes *ad hoc*. The Peri-stimulus time histograms (PSTHs) was calculated with 100ms bins (over 20 repeated trials). Thresholds were defined using both the amplitude and duration of responses. For increases in firing rate, this was defined as the pre-stimulus baseline +3 standard deviation (SD) with 100ms duration, while for decreases in firing rate this was the pre-stimulus baseline −1 SD with 300ms duration. Baseline is defined as the average firing rate in the 10 s preceding the stimulus over 20 repeated trials. We next defined a set of rules to assign each neuronal response to a specified class. The rationale behind these rules was based on translating the human observer knowledge into rules and codes and to avoid black box classification system that has only clear inputs and outputs and no knowledge about system parameters.

We therefore defined three time spans: 1) Duration of time during the light stimulus (ON period), 2) Duration of time directly following the light stimulus and equal to it (OFF period), 3) Duration of time beyond the OFF period (Sustained period) which was defined arbitrarily as 6 times the ON period. For this dataset the values are 1 s, 1 s and 6 s respectively. Neuronal responses were analyzed within

each of the above-mentioned time spans to see if the firing rate crossed these thresholds compared to the pre-stimulus baseline. Then a look up table created for these rules and results has been defined to assign each neuron to a class. The rules are as shown in Table S2 for 10 separate categories.

Accordingly, single units could be classified into 10 categories. ‘ON’ cells demonstrated a significant increase in firing rate after light onset that returned to baseline during the light pulse. ‘On Suppressed by Dark’ units showed a significant increase in firing rate during the light pulse and then a decrease in firing rate below baseline following light offset. ‘ON-OFF’ units showed a significant increase in firing rate at light onset and again at light offset. Slow Sustained’ showed a significant increase in firing rate during the light pulse which remained elevated following light offset and into the sustained period. ‘OFF’ units showed an increase in firing rate following cessation of the light pulse. ‘OFF Suppressed by Light’ units showed a significant decrease in firing rate during the stimulus followed by a rapid increase in firing rate at light offset. ‘Suppressed ON’ and ‘Suppressed OFF’ units showed a significant decrease in firing rate at light onset and offset, respectively. ‘Suppressed ON-OFF’ units showed a significant decrease in firing rate at both light onset and offset. ‘Non Light Responsive’ units were classified as units whose firing rate did not cross the threshold during any of the three defined time spans. Single units were additionally checked manually to verify the classification of the response.

Latency & amplitude analysis

Amplitude and Latencies were calculated for individual light responsive units. Amplitude is defined as the change in firing rate between light onset/offset and peak response. Latency is the time difference between stimulus onset/offset and peak response. Latencies were calculated from smoothed PSTHs with 10ms bin to retain an appropriate time resolution, while amplitude was calculated with a 100ms bin. Latency was then plotted for different component of light-responsive units based upon their classification to the 1 s light pulse as above and binned at 10ms. Latency to the ON component of responses (ON, On Suppressed by Dark and ON-OFF units) was calculated as the time at which maximum firing rate was reached following light onset. Latency to the OFF component of responses (OFF, OFF Suppressed by Light and ON-OFF units) were calculated as the time at which maximum firing rate was reached following light offset.

Sensitivity analysis

Single units were filtered to ensure that the firing rate at the highest irradiance demonstrated a significant change in firing rate that was > 3 SD above the pre-stimulus baseline. If this criterion was met, the response of that unit at the five lower intensities was used for analysis regardless of whether it crossed the confidence interval. Sensitivity curves were calculated by subtracting the pre-stimulus baseline from the average firing rate over the first 400ms of the 1 s light step.

Light avoidance

Mice were kept under ambient light conditions and pupil dilation was not performed. Mice were placed in a newly built arena, 45 × 39 × 33 cm, with dark and light chambers of the same size, connected by an aperture, 6 × 7 cm, that allowed animals to transition freely between the chambers. The light chamber was illuminated by an LED light projector on the top of the chamber, emitting 300 lux at the arena floor. Before every test, both chambers were thoroughly cleaned with 70% ethanol. After placing a mouse in the light chamber close to the aperture, and in the center of the arena, camera recording was started, and the LED light projector was turned on. Each trial lasted 10 minutes for each mouse and the animals were naive to the test (i.e., single trial per mouse). A mouse was considered to have crossed into a different chamber when three paws had crossed to that chamber. Data was recorded by a digital camera and analyzed using AnyMaze software. The arena was defined at the time of analysis, with each compartment being outlined. The ellipsoids seen in each compartment outline its central area. Light avoidance was measured by the percentage of total time that was spent in the dark chamber. Tracking plots were created showing the movement of each mouse inside the arena; *Blue dot* shows the starting position and *Red dot* the end position. Heatmaps show the mean occupancy of the arena for each group (calculated by averaging the time spend in each location, across all mice from each group, and dividing it by the number of tests). Maximum occupancy was set for 20 s (if average time in a given position was greater than 20 s it is still shown in red).

QUANTIFICATION AND STATISTICAL ANALYSIS

All values are presented mean ± SEM (standard error of the mean) unless otherwise stated; N, number of animals, retinas or independent experiments performed, where appropriate; n, number of retinas, images or retinal organoids examined, where appropriate. Statistical significance was assessed using Graphpad Prism software and denoted as $p < 0.05$ = *; $p < 0.01$ = **; $p < 0.001$ = ***. Appropriate statistical tests were applied including 2 tailed t test (Mann Whitney), 1-way ANOVA with Tukey’s correction for multiple comparisons, 2-way ANOVA with Bonferroni’s correction, and Paired and unpaired t tests were used to compare latency and amplitude calculations. Figures were generated in either CorelDraw 2020 (V22.1.0.517, Corel Corporation) or Adobe Photoshop.

Cell Reports, Volume *35*

Supplemental information

Restoration of visual function in advanced disease after transplantation of purified human pluripotent stem cell-derived cone photoreceptors

Joana Ribeiro, Christopher A. Procyk, Emma L. West, Michelle O'Hara-Wright, Monica F. Martins, Majid Moshtagh Khorasani, Aura Hare, Mark Basche, Milan Fernando, Debbie Goh, Neeraj Jumbo, Matteo Rizzi, Kate Powell, Menahil Tariq, Michel Michaelides, James W.B. Bainbridge, Alexander J. Smith, Rachael A. Pearson, Anai Gonzalez-Cordero, and Robin R. Ali

SUPPLEMENTARY INFORMATION

Supplementary Figures

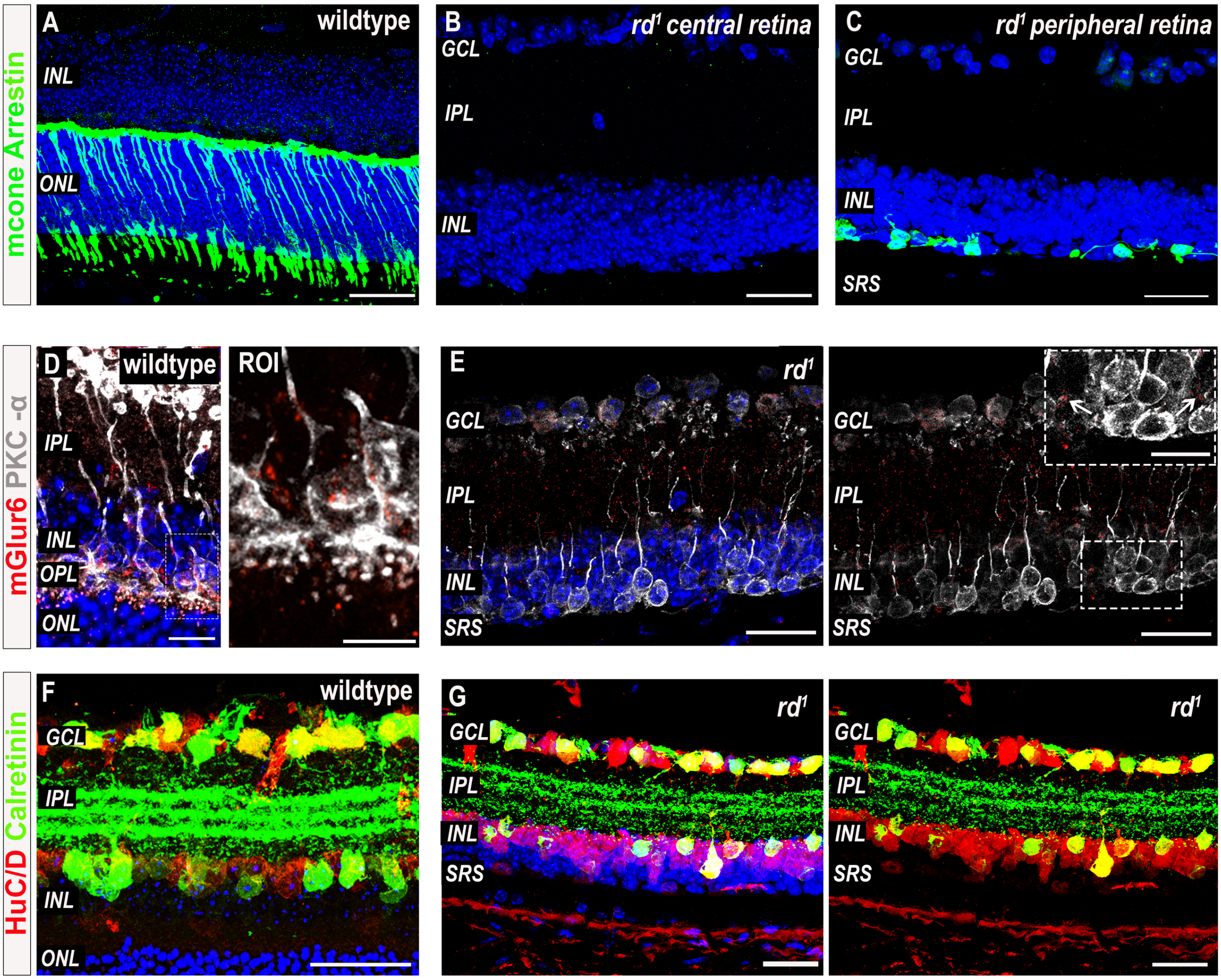

Supplementary Figure S1. Characterization of 3-month old *rd1/Foxn1^{nu}* retina. Related to Figures 1 and 3 Representative confocal images of immunohistochemistry for retinal cell markers (N ≥ 3 animals). **(A)** 3-month old wildtype retina showing typical cone morphology. **(B)** Age-matched *rd^1/FoxN1^{nu}* (*rd^1*) retina. The majority of the retina did not show presence of any cone photoreceptor cells. Image shows central retina. **(C)** Small, typically peripheral, regions of the *rd^1* retina retained a few disorganized Cone Arrestin+ (*green*) cones. Image shows mid-peripheral retina. **(D)** Wildtype retinas display widespread punctate labelling for mGluR6 (*red*) in the OPL. This colocalised with the PKC-a+ (*grey*) post-synaptic processes of rod bipolar cells (ROI, *right*). **(E)** In the *rd^1*, bipolar cells displayed a significantly reduced dendritic arborization. While some mis-localized mGluR6 staining was visible in the IPL, little or no mGluR6 positive structures were found between the INL and SRS (insert). **(F)** Wildtype retina showing morphology and distribution of amacrine and ganglion cells in the retina. **(G)** In the *rd^1*, amacrine cells had unusual morphology, as they appeared fewer and smaller. Some disorganization and reduction in staining for calretinin positive processes was visible in the IPL. Ganglion cells displayed typical morphology. Scale bar 25µm, except inner panel in E, 12.5 µm). GCL – ganglion cell layer, IPL – inner plexiform layer, INL – inner nuclear layer, OPL – outer plexiform later, ONL – outer nuclear layer, SRS – subretinal space).

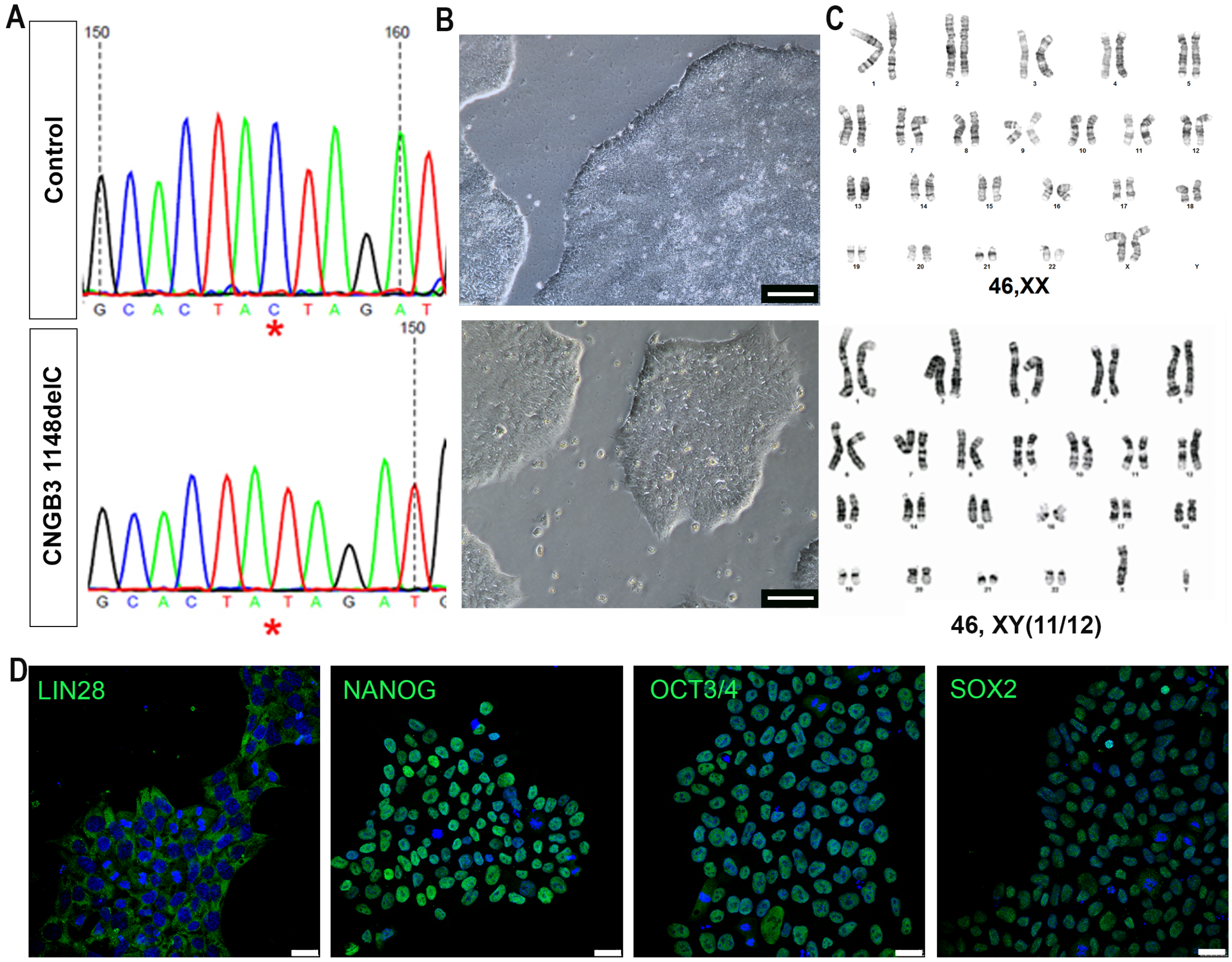

Supplementary Figure S2. Characterisation of CNGB3 hiPSC line. Related to Figure 1. PBMCs from CNGB3 patient was reprogrammed using episomal methods. **(A)** Sequencing data of the WT hESC line and the CNGB3 hiPSC line showing the existence of a single base pair deletion in the CNGB3 hiPSC line (*red asterisk*). **(B)** Representative bright field image of both cell lines showing typical pluripotent cell colonies morphology. **(C)** A normal karyotype was observed in both lines. **(D)** Representative examples of immunostaining showing that the CNGB3 cell line was positive for all pluripotency markers tested. Scale bars 100µm, B; 25µm, D.

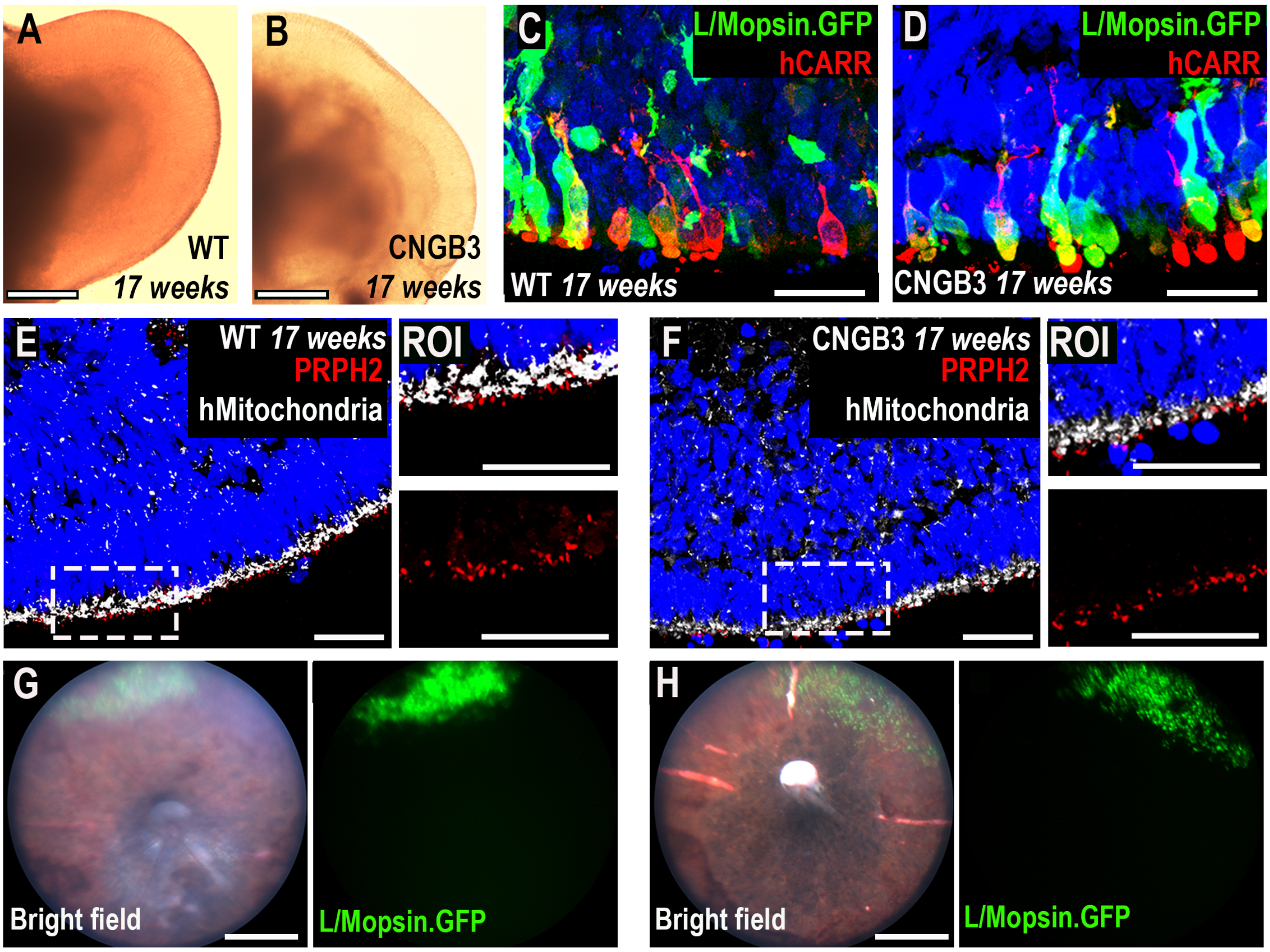

Supplementary Figure S3. Differentiation and transplantation of cone photoreceptors from WT and CNGB3 PSC lines. Related to Figures 1 and 2. **(A)** Representative bright-field image of WT hESC-derived retinal organoid at week 17 of differentiation. **(B)** Representative bright-field image of CNGB3 hiPSC-derived retinal organoid at week 17 of differentiation displaying very similar morphology. **(C, D)** Human CONE ARRESTIN+ (*red*) cells were detected by 17 weeks of differentiation and displayed a similar morphology in WT and CNGB3 derived organoids. **(E, F)** At 17 weeks, organoids derived from both cell lines show presence of a mitochondria-enriched (*grey*) IS-like region. Small PRPH2+ (*red*) buds were present in both WT and CNGB3 derived organoids and these were correctly located distal to mitochondria-rich IS. **(G-H)** Representative examples of L/MOpsin GFP+ cones in both WT **(G)** and CNGB3 **(H)** organoids. **(I)** proportion of total cells that were GFP+ at 17weeks of differentiation in organoids derived from WT and CNGB3 cell lines, as determined by FACS. **(J-K)** Representative fundoscopy images showing that, following transplantation, the GFP+ WT cones **(J)** and CNGB3 cones **(K)** survive similarly in the subretinal space, up to at least 12 weeks post- transplantation. **(L)** Low power montage showing extent of spread of transplanted GFP+ WT cones. Scale bars, 70µm, A-B; 25µm, C-F; 75µm, J-K.

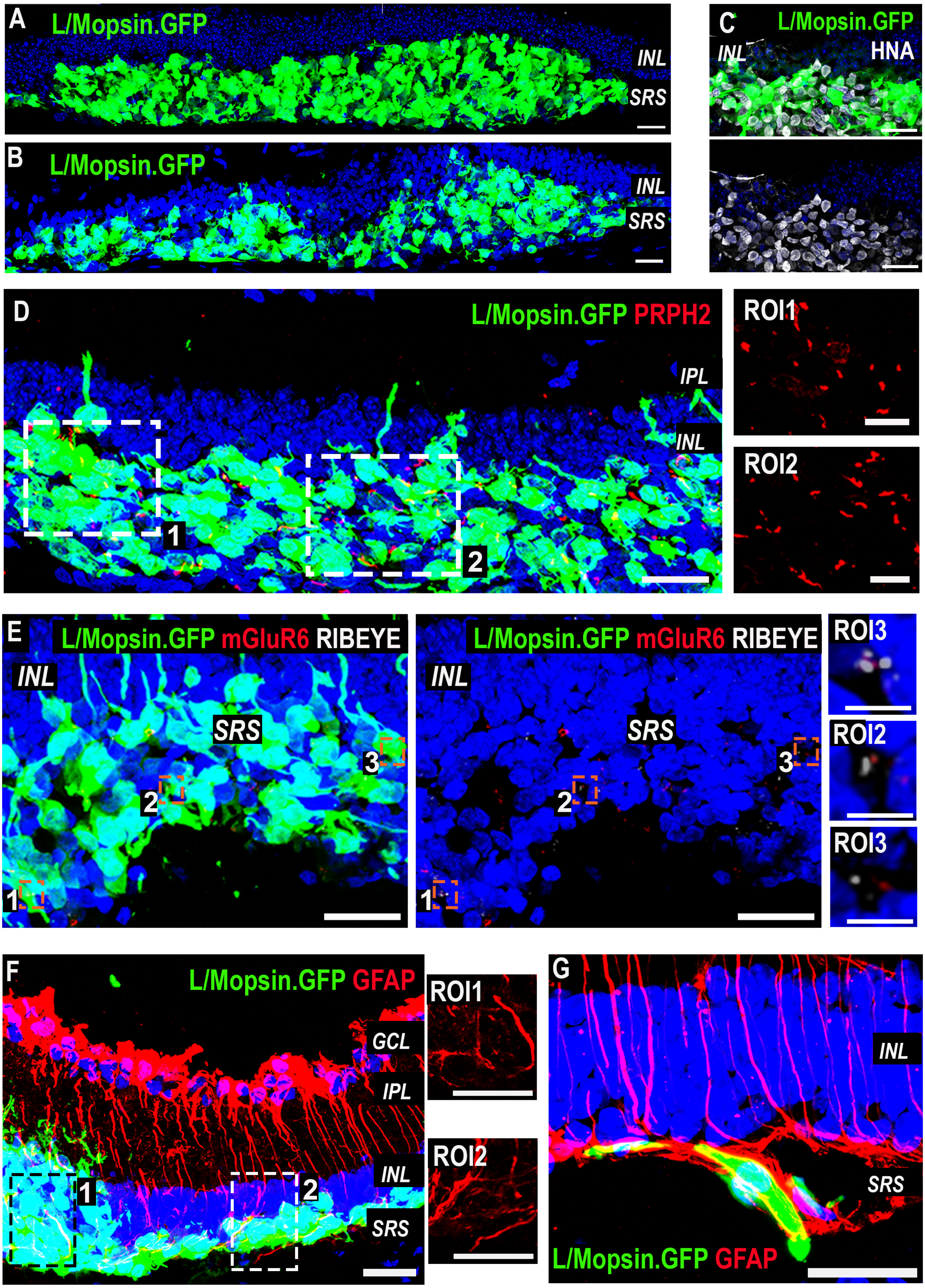

A
L/Mopsin.GFP
INL
SRS
B
L/Mopsin.GFP
INL
SRS
C
L/Mopsin.GFP
HNA
INL
D
L/Mopsin.GFP PRPH2
IPL
INL
1
2
ROI1
ROI2
E
L/Mopsin.GFP mGluR6 RIBEYE
INL
SRS
1
2
3
L/Mopsin.GFP mGluR6 RIBEYE
INL
SRS
1
2
3
ROI3
ROI2
ROI3
F
L/Mopsin.GFP GFAP
GCL
IPL
INL
SRS
1
2
ROI1
ROI2
G
INL
SRS
L/Mopsin.GFP GFAP

Supplementary Figure S4. Transplanted WT and CNGB3 cones mature in the *rd1* retina. Related to Figures 1-3. Representative confocal images of immunohistochemistry for retinal cell markers. Representative confocal images of immunohistochemistry for retinal cell markers. **(A, B)** Representative images of dense GFP+ cell mass following transplantation of **(A)** WT- and **(B)** CNGB3-derived cones. **(C)** CNGB3 cones survived in the subretinal space (SRS) and expressed human nuclear antigen (HNA; *grey*) (bottom). **(D)** CNGB3 cones matured *in vivo*, presenting PRPH2+ segment-like structures (ROI1, ROI2). **(E)** Pre-synaptic RIBEYE (grey) and post-synaptic mGluR6 (red) proteins were found in close proximity to one another in *rd1* mice transplanted with CNGB3 cones (ROI1-3). **(F, G)** GFAP+ (*red*) host Müller glial processes extended into the cell mass. No glial scar was observed (ROI1, ROI2). Scale bar 25μm, except ROIs in E 6.25μm. GCL – ganglion cell layer, IPL – inner plexiform layer, INL – inner nuclear layer, SRS – subretinal space.

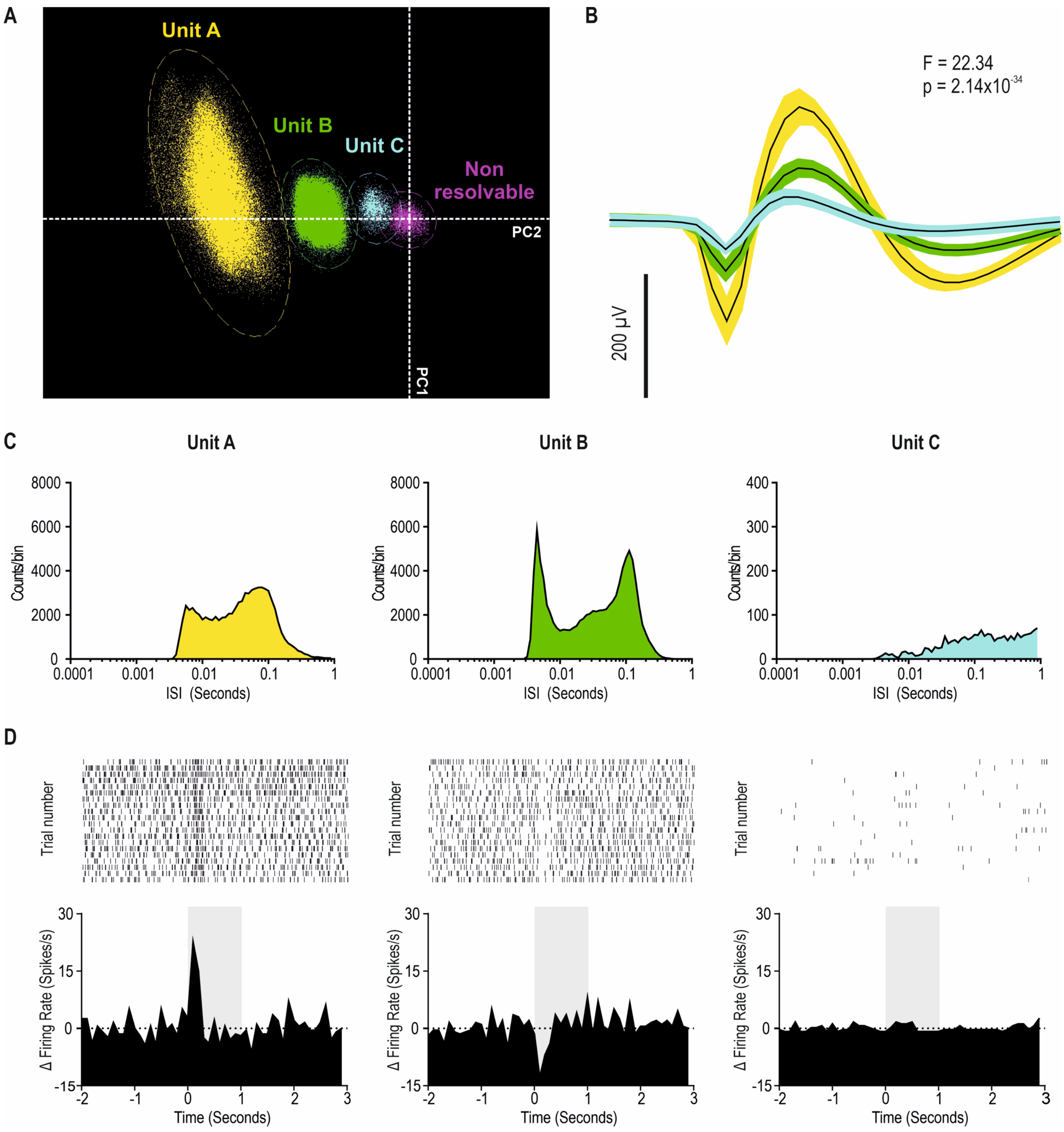

Supplementary Figure S5. Analysis of Multi Electrode Array (MEA) data. Related to Figure 6 **(A)** Spike waveforms in a 2D representation of principal component space from a representative channel from *rd1* retina treated with WT cones (X axis = PC1; Y axis = PC2) shows three identifiable single units (Unit A = Yellow; Unit B = Green; Unit C = Blue; Non-resolvable spikes = Purple). **(B)** Unit A, B and C show differences in spike shape and amplitude (mean ± SD). High Manova F values and low P-values indicate good cluster separation for isolated single units. **(C)** Log Inter-spike Interval histograms of Unit A (yellow), B (green) and C (blue) show peaks at discrete intervals greater than 1ms, indicative of spikes fired in bursts. **(D)** (*top*) Raster plot and *(bottom)* average PSTH show responses of the single units isolated in *(A)*. Unit A =Transient ON, Unit B = Suppressed ON and Unit C = non-light responsive when presented with 20 repeats of a 1s light pulse from darkness and a 10s ISI (grey bar indicates light pulse; irradiance = 4.21×10^{15} photons$\cdot$cm$^{-2}\cdot$s^{-1}).

A

Response Categorisation	Response Profile	*Gnat1*$^{-/-}$	*rd*^{1} + *WT cones*
ON	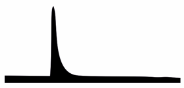	10.3	25.9
On Suppressed by Dark	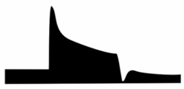	11.3	2.5
ON-OFF	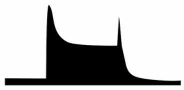	47.8	6.4
Slow Sustained	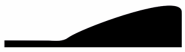	0.0	1.2
OFF	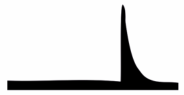	8.7	3.8
OFF Suppressed by Light	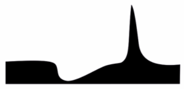	11.9	1.7
Suppressed ON	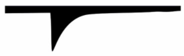	0.9	6.4
Suppressed OFF	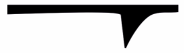	1.4	1.6
Suppressed ON-OFF	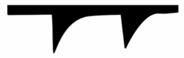	3.8	0.0
Non Light Responsive		3.9	50.5

B

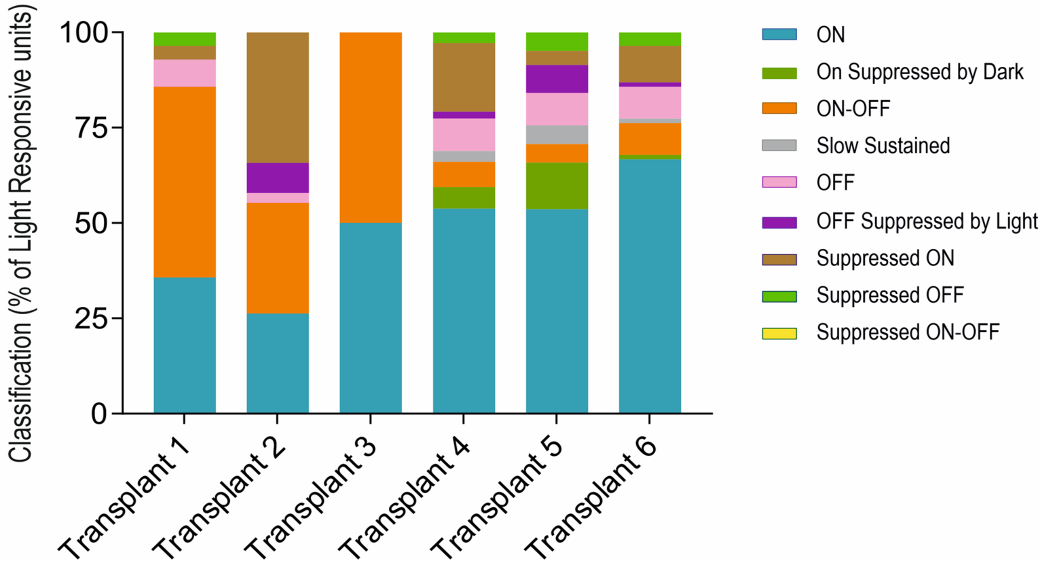

Supplementary Figure S6: Reproducibility of light-responses in *rd1/FoxN1^{nu}* treated with WT cones. Related to Figure 6. **(A)** % (mean) response type of single units for *rd1/FoxN1^{nu} (rd^{1})* retinas transplanted with WT cones (N = 6 retinas; n = 687 single units) and *Gnat1$^{-/-}$* (N = 5; n = 1031) mice. **(B)** A similar variety of light response types were seen in each of the six *rd^{1}* retinas transplanted with WT cones (Total light-responsive units = 340; N = 6).

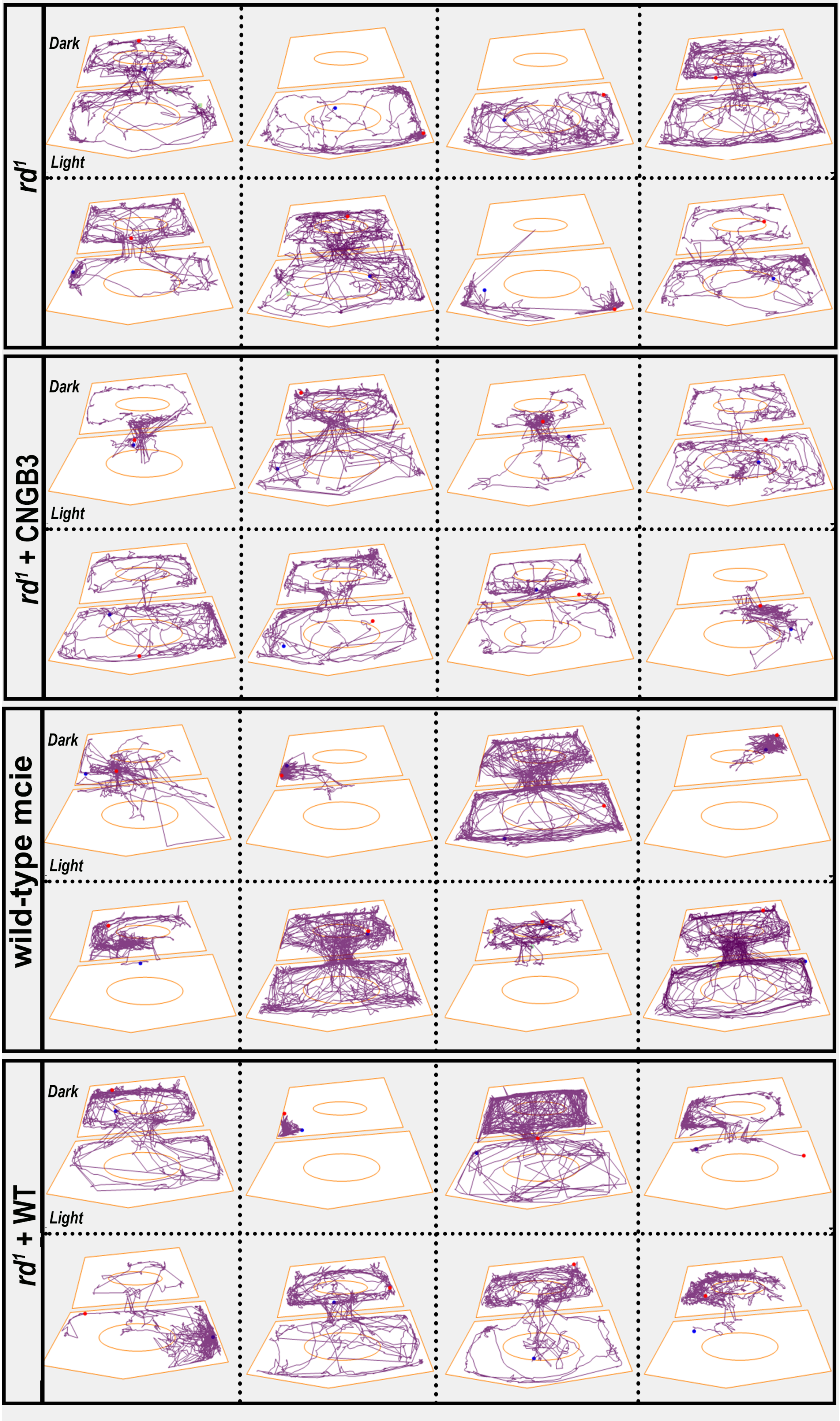

rd1
Dark
Light
rd1 + CNGB3
Dark
Light
wild-type mcie
Dark
Light
rd1 + WT
Dark
Light

Supplementary Figure S7. Transplantation of WT cones restores light avoidance behaviour in *rd^1* mice. Related to Figure 7. Further representative examples, chosen randomly, of tracking plots from different animals in each experimental group. Blue dot represents each animal's position at the start of the recording and red dot the position at the end of the recording. Ellipsoids (I-L) define the central area of each compartment. Untreated *rd^1* animals and those receiving non-functional CNGB3 cones displayed a similar pattern of movement, with most mice exploring both light and dark compartments equally. In contrast, *rd^1* animals transplanted with WT cones showed a clear preference for the dark compartment of the arena. Most *rd^1* animals transplanted with WT cones showed similar exploration pattern to normal wildtype animals.

Supplementary Tables

**Animal**	***Gnat1*** $^{-/-}$	***rd1*** **+ WT cones**	***rd1*** **+ CNGB3 cones**	***rd1***
**Number of recorded retinas**	5	6	5	5
**Transplanted cell line**	N/A	hESC H9	hiPSC CNGB3	N/A
**Number of transplanted cones per retina**	N/A	450-500k	450-500k	N/A
**Mouse age at transplantation**	N/A	3 months	3 months	N/A
**Mouse age at recording**	6 months	6 months	6 months	6 months
**Number of recorded Electrodes**	600	540	480	600
**mERG**	560	150	0	0
**Single Units**	1031	687	945	641
**Light Responsive**	991	340	8	13
**Non Light Responsive**	40	347	937	628

Supplemental Table S1. Summary of MEA recordings in all experimental groups. Related to Figures 4, 5 & 6. Table summarises the key information from Multi Electrode Array recordings in each experimental group: the number of retinas recorded, the age at transplantation (if applicable), the cell line transplanted (if applicable), the number of cells transplanted (if applicable), age of retina at time of recording, number of electrodes recorded, number of electrodes with positive mERG, number of single units resolved after spike sorting, and the number of those units which were light-responsive or non-light responsive following classification.

Response Categorisation	ON	OFF	ON Suppressed	OFF Suppressed	Sustained
**Type 1**<br>ON	1	0	0	0	0
**Type 2**<br>On Suppressed by Dark	1	0	0	1	0
**Type 3**<br>ON-OFF	1	1	0	0	0
**Type 4**<br>Slow Sustained	1	1	0	0	1
**Type 5**<br>OFF	0	1	0	0	0
**Type 6**<br>OFF Suppressed by light	0	1	1	0	0
**Type 7**<br>Suppressed ON	0	0	1	0	0
**Type 8**<br>Suppressed OFF	0	0	0	1	0
**Type 9**<br>Suppressed ON-OFF	0	0	1	1	0
**Type 10**<br>Non light responsive	0	0	0	0	0

Supplementary Table S2. Summary of rules used to classify neural responses into one of 10 categories. Related to STAR Methods, section *Identification of Light Responses*. Single units were defined as light responsive when their firing rate either increased (+3SD) or decreased (-1SD) when compared to the pre-stimulus baseline. The time at which neuronal activity crossed the threshold in response to the 1s light step was defined in three timeframes: 1) Duration of the light stimulus (1s; ON period), 2) Duration of time directly following the light stimulus and equal to it (1s; OFF period), 3) Duration of time beyond the OFF period (Sustained period) which was defined as 6 times the ON period. When single unit responses cross the threshold within the specified frame, the expression value is 1 (True) or 0 (False), and generated a look up table which classified the ten response categories. When the response crosses the threshold in the specified time span, the expression value becomes 1 (True) or 0 (False).
